# Natural Products from Medicinal Plants against Phytopathogenic *Fusarium* Species: Current Research Endeavours, Challenges and Prospects

**DOI:** 10.3390/molecules26216539

**Published:** 2021-10-29

**Authors:** Hlabana A. Seepe, Winston Nxumalo, Stephen O. Amoo

**Affiliations:** 1 Agricultural Research Council—Vegetables, Industrial and Medicinal Plants, Roodeplaat, Private Bag X293, Pretoria 0001, South Africa; 2 Department of Chemistry, University of Limpopo, Private Bag X1106, Sovenga, Polokwane 0727, South Africa; 3 Indigenous Knowledge Systems Centre, Faculty of Natural and Agricultural Sciences, North-West University, Private Bag X2046, Mmabatho 2735, South Africa; 4 Department of Botany and Plant Biotechnology, Faculty of Science, University of Johannesburg, P.O. Box 524, Auckland Park 2006, South Africa

**Keywords:** *Fusarium*, medicinal plants, antifungal, isolated compounds, extracts, essential oils, crop diseases

## Abstract

Many *Fusarium* species are pathogenic, causing crop diseases during crop production and spoilage of agricultural products in both commercial and smallholder farming. *Fusarium* attack often results into food contamination, yield loss and increases in food insecurity and food prices. Synthetic fungicides have been used as a control strategy for the management of crop diseases caused by *Fusarium* pathogens. The negative effects associated with application of many synthetic pesticides has necessitated the need to search for alternative control strategies that are affordable and environmentally safe. Research on medicinal plants as control agents for *Fusarium* pathogens has received attention since plants are readily available and they contain wide variety of secondary metabolites that are biodegradable. The activities of solvent extracts, essential oils and compounds from medicinal plants have been tested against *Fusarium* phytopathogenic species. A summary of recent information on antifungal activity of plants against *Fusarium* species is valuable for the development of biopesticides. This paper reviews the antifungal research conducted on medicinal plants against *Fusarium* pathogens, over a 10-year period, from January 2012 to May 2021. We also highlight the challenges and opportunities of using natural products from medicinal plants in crop protection. Several databases (Science Direct and Web of Science) were used to obtain information on botanical products used to control *Fusarium* diseases on crops. Keywords search used included natural products, antifungal, *Fusarium*, crops diseases, phytopathogenic, natural compounds and essential oil.

## 1. Introduction

The genus *Fusarium* is among the largest fungal genera consisting of pathogenic and non-pathogenic species [1]. Although discovered over more than 200 years ago, the genus remains taxonomically complex [2]. The pathogenic *Fusarium* species are well known to consist of agriculturally important crop pathogens, mycotoxin producers and opportunistic human pathogens [3]. The members of this genus have been isolated from plant materials and soil as pathogens, ascomycetes, endophytes and saprobes [4,5]. Various members of *Fusarium* genus are known to cause diseases in crops, including maize, wheat, rice, potatoes, tomatoes, beans, sorghum, banana, sugar cane, mangoes and other economically important crops [6]. The growth and development of *Fusarium* pathogens depend on factors such as seasons, climatic conditions (temperature and humidity) and geographical locations [7,8].

*Fusarium* fungal pathogens such as *F. graminearum*, *F. moniliforme*, *F. oxysporum* and *F. verticillioides* are known to infect cereal crops, fruits and vegetables (Table 1). They cause diseases that include head or seed blights, vascular wilts, pokkah boeng, bakanae, panama disease, stem, ear, crown and root rots [9,10,11,12,13,14]. The diseases can cause devastating economic yield loss in the field and during post-harvest storage, and result in a greater impact on food insecurity. *Fusarium* species are also more prevalent and major causes of quality deterioration of fruit and vegetables. *Fusarium* diseases may initiate in the roots from soil-borne spores/inoculum or on the above-ground parts of the crop, introduced through air, water or agricultural equipment [15,16]. The pathogens can also infect crops via injuries made by emerging roots, insects, nematodes and other environmental factors, resulting in disease symptoms such as wilting, necrosis and chlorosis [17,18].

The economic damage caused by *Fusarium* species is through their direct attack of crops in the fields and by the production of allergenic compounds and mycotoxins, which contaminate commodities during post-harvest storage. They produce fungal secondary metabolites such as deoxynivalenol, nivalenol, diacetoxyscirpenol, zearalenone, fusaric acid and fumonisins, all of which are harmful to humans and livestock [19,20,21,22,23]. Mycotoxin contamination is a major food safety concern in many parts of the world, with an estimate of almost 25% of the world’s crops being affected [24,25]. Consumption of food products contaminated with mycotoxins is associated with health risks such as oesophageal cancer, carcinogenesis, mutagenicity and neural tube defects [26,27]. The world’s population is estimated to increase to more than 10 billion by 2050, and this will put more pressure on farmers to produce more nutritious and safe food [28]. On the other hand, climate change, drought, pests and diseases remain major factors affecting current food production systems [28,29].

Due to the economic impact of crop diseases in agriculture and the effect of mycotoxins on food safety and international trade, the *Fusarium* genus remains the focus of many studies [24]. It is clear that *Fusarium* crop diseases cause deleterious effect on crop production and quality; therefore, effective and safe control measures that are sustainable must be developed and implemented. An estimated 2 billion people amounting to a quarter of the global population were affected by moderate-to-severe food insecurity in 2019 [30], a condition that has been exacerbated by the recent global coronavirus pandemic. Thus, discovering sustainable, safe and effective control strategies for controlling crop diseases remains imperative towards achieving the second goal, amongst others, of the Sustainable Development Goals (SDGs), which is ‘’to end hunger, achieve food security and improved nutrition and promote sustainable agriculture’’. This review provides an overview of current research activities from 2012, as well as the challenges and prospects of developing natural products from medicinal plants as a source of biopesticides to control phytopathogenic *Fusarium* species against the backdrop of using synthetic chemicals.

**Table 1:** The most common *Fusarium* species known to infect cereal crops, fruits and vegetables.

Pathogen	Crop	Common Disease	Reference
*Fusarium acuminatum*	kiwifruit	post-harvest rot	[31]
*Fusarium asiaticum*	soybean	head blight or ear rot	[32]
*Fusarium avenaceum*	wheat, beans, maize	head blight or ear rot	[33,34,35]
*Fusarium boothii*	wheat, maize	head blight or ear rot	[36]
*Fusarium crookwellense*	wheat, potatoes	ear rot, head blight, dry rot	[37,38]
*Fusarium culmorum*	wheat	seedling blight, ear blight, stalk rot	[35]
*Fusarium equiseti*	wheat, barley	crown rot, damping-off	[39]
*Fusarium falciforme*	bean	wilt disease, necrosis	[40]
*Fusarium fujikuroi*	rice	bakanae disease	[41]
*Fusarium graminearum*	wheat, corn	*Fusarium* head blight	[35]
*Fusarium kuroshium*	avocado tree	*Fusarium* dieback	[42]
*Fusarium kyushuense*	tobacco	*Fusarium* wilt	[43]
*Fusarium langsethiae*	oats, wheat, barley	*Fusarium* head blight	[44]
*Fusarium nivale*	wheat, rye	seedling blight, *Fusarium* head blight	[45]
*Fusarium nygamai*	corn, rice, sorghum, bean, cotton	seedling blight, foot rot	[46]
*Fusarium oxysporum*	Tomato, cucumber, watermelon	vascular wilt	[47]
*Fusarium poae*	wheat	*Fusarium* head blight	[33,34,35]
*Fusarium proliferatum*	wheat, maize, onion, soybean	necrotic leaf, bulb rot, root rot, ear rot diseases	[48,49,50]
*Fusarium sambucinum*	potato	sprout rot, dry rot	[51]
*Fusarium semitectum*	pineapple, okra, bitter gourd, cucumber, green chill	fusariosis, fruit rot	[52,53]
*Fusarium solani*	peas, soybean, beans, potatoes	stem rot, stem rot, dry rot	[54]
*Fusarium sporotrichioides*	wheat, cereals	*Fusarium* head blight	[55]
*Fusarium subglutinans*	maize, mango, pineapple, pine, sorghum	pitch canker,	[56,57]
*Fusarium sulphureum*	potato	dry rot	[58,59]
*Fusarium thapsinum*	sorghum, banana, maize, peanut, soybean	stalk rot	[60]
*Fusarium tricinctum*	cereal	root rot disease, *Fusarium* head blight	[61,62]
*Fusarium verticillioides*	maize, wheat, corn	ear and stalk rot	[63,64,65,66,67]

## 2. Environmental and Health Implications of *Fusarium* Control in Crop Production Using Synthetic Chemicals

**Figure 1:**
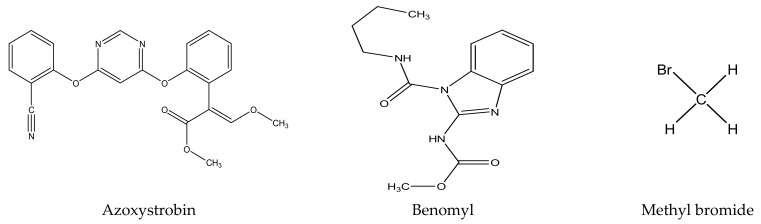

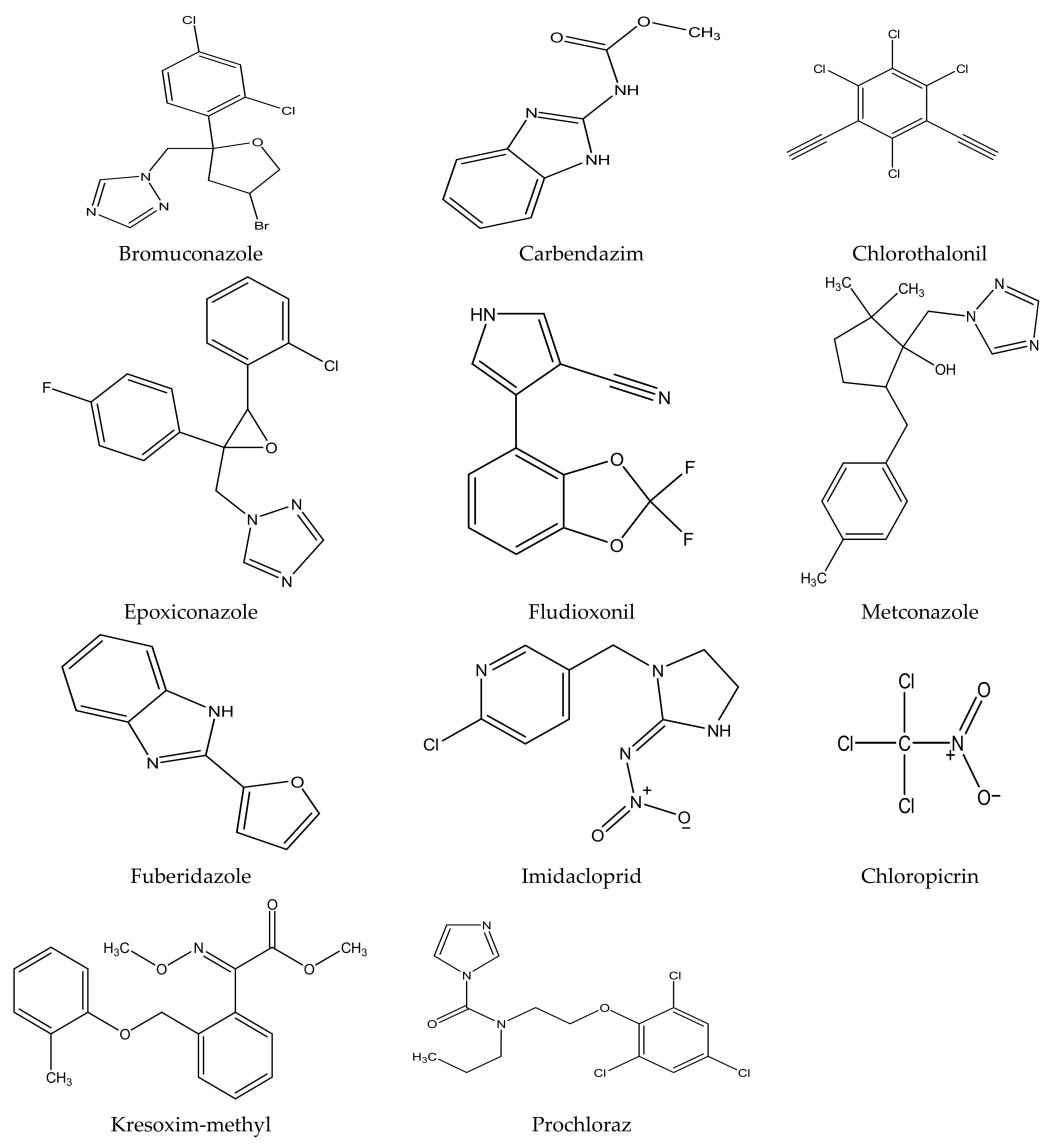

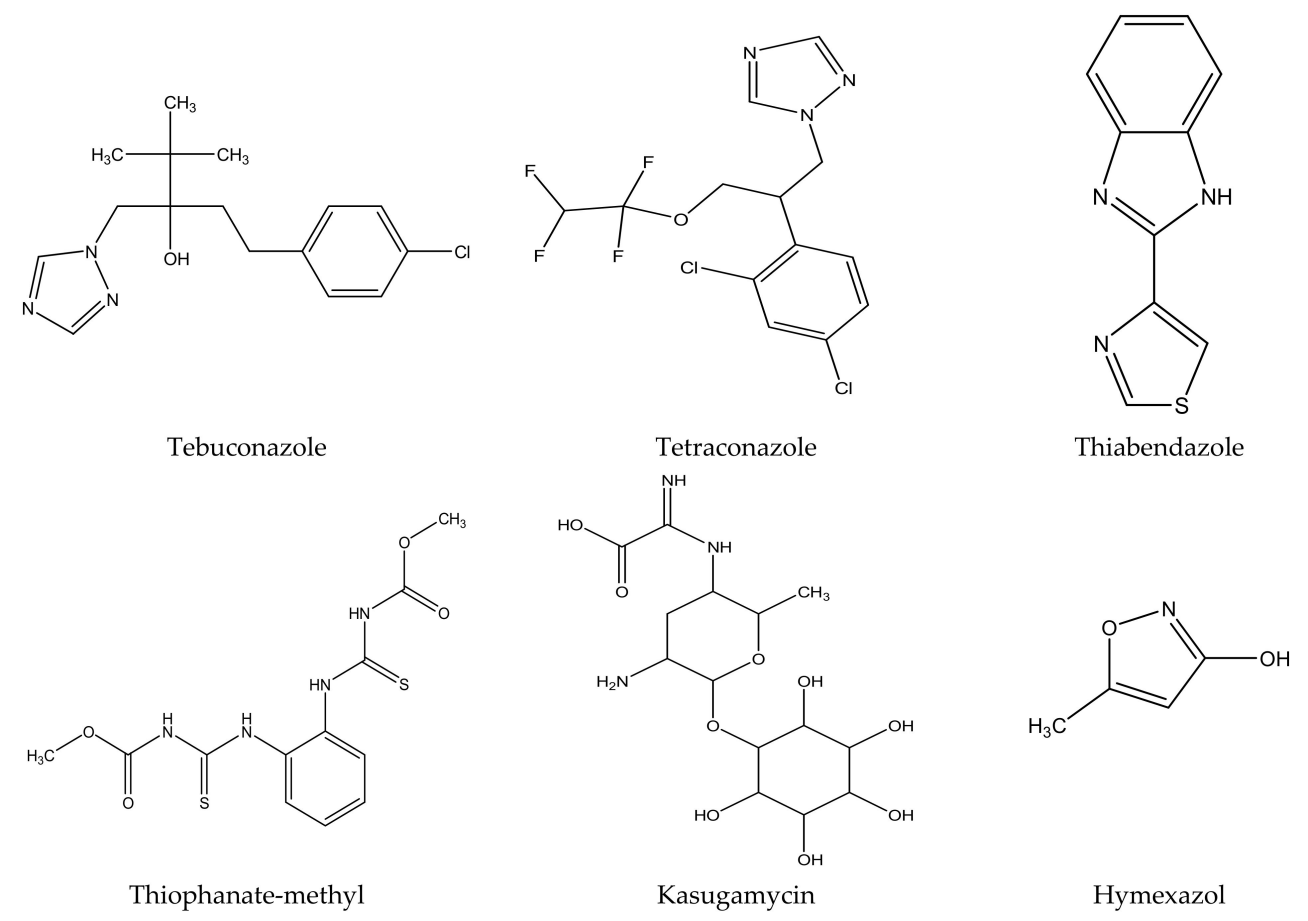
Conventional synthetic fungicides used to control crop diseases caused by phytopathogenic *Fusarium* species.

There are several strategies already used in crop production to control crop diseases caused by *Fusarium* species [68,69]. Historically, the application of synthetic pesticides remains the primary strategy to control diseases, which have benefited commercial farmers since the first fungicides were introduced in the 1800s. Random chemical synthesis and evaluation of the activity against phytopathogenic species has resulted in many agrochemicals in different parts of the world. The introduction of synthetic pesticides has reduced the effect of many crop diseases in agricultural production including those that are caused by *Fusarium* pathogens, and it remains a key component of disease management worldwide, particularly in developing countries [69,70]. Chemical control methods are preferred in commercial crop production due to their effectiveness to also control soil-borne crop pathogens and the availability of spraying technology for easy application. Figure 1 presents the structures of few synthetic fungicides used to control *Fusarium* pathogens [35,47,71,72,73,74,75,76]. The chemicals were formulated to be applied as fruit and seed treatments, fumigants or in foliar applications.

Although synthetic fungicides have benefited crop production for decades, nowadays, the use of such chemicals is restricted or discouraged for several reasons. The overapplication or misuse of synthetic fungicides has raised serious concerns including their impact on the environment, contamination of drinking water and the effect on human health and livestock [77,78,79,80,81]. Generally, pesticides are known to affect soil microorganisms (often the untargeted species), and sometimes lead to an imbalance in the ecosystem [82,83,84]. The application of methyl bromide in the soil was a common sterilization practice in agriculture to control *Fusarium* species and other soil pests [85]. Methyl bromide was used as a versatile, single treatment and long-lasting soil fumigant with relatively no soil residue to sterilize soil before planting, as it controlled weeds, nematodes and almost all living organisms in the soil [86,87]. Being a very volatile gas, it usually ends up in the air causing smog as well as thinning of the protective ozone layer in the stratosphere [86,87]. Methyl bromide is categorized as a substance that causes ozone layer damage [85] and its use is banned under the Montreal Protocol international treaty to protect the ozone layer [86,87] Methyl bromide is also toxic and several studies have indicated its neurological effects in humans and resultant severe lung injuries [85].

Apart from environmental and human health challenges as a result of synthetic fungicides, farmers have been struggling with emergence of resistance against some commonly known fungicides since the 1970s [88,89]. As an example, thiabendazole, which was one of the most effective fungicides against a wide variety of pathogens, is no longer an effective treatment. However, some farmers are still using it in combination with other chemicals to control dry rot diseases. Carbendazim is another kind of fungicide that is no longer readily available on the market due to resistance concerns, and this fungicide is believed to be banned in some countries including in the European Union (EU) countries [90,91,92]. Fungicide poisoning to farmers is a common problem in many countries, especially in developing countries [93,94,95]. Although the World Health Organization (WHO) has regarded fludioxonil as a pesticide that does not cause hazard in normal use, its manufacturer specified that fludioxonil is moderately toxic against *Oncorhynchus mykiss* (Rainbow trout), daphnia and other aquatic invertebrates [96,97,98].

Other fungicides such as chloropicrin do not persist in the environment for a long period of time; however, vapour or toxic gases produced during decomposition of chloropicrin can cause severe headaches, pulmonary oedema and may have adverse effects on the nervous system [99]. Fungicides in the azole chemical class such as benzimidazoles are very successful in the treatment of many crop diseases worldwide [100]; however, they are predisposed to the emergence of resistance by crop pathogens. Nowadays, in order to minimize or delay resistance, azole fungicides are usually applied as a mixture with other fungicides such as benomyl [101]. However, it is noteworthy that the use of benomyl has been restricted in Sweden and New Zealand since 1982 [99]. On the other hand, the WHO justified benomyl as a moderately safe fungicide against mammals, whilst other international institutions in the United States of America have categorized benomyl as a teratogenic and carcinogenic chemical [99]. All these challenges have negatively affected the market and availability of fungicides used to control crop diseases, mostly in commercial farming. Additionally, synthetic fungicides are not recommended for application in organic farming system, and consumers are willing to pay more for food or crops that are produced organically [102]. This already demarcates the society and puts more financial pressure on the poorest; hence, there has been an increase in food insecurity. Furthermore, synthetic fungicides are not readily available and/or affordable to small-holder farmers. This kind of farming is largely practiced in poor resourced communities; however, it is still a source of food and income generation for many households [103,104].

In small-holder farming, crops and vegetables are in most cases collected and consumed upon harvest. After harvest, the surplus grains and vegetables are stored and consumed during the off-season. This practice makes it impractical to apply synthetic fungicides both in the field and during post-harvest storage. To make matters worse, synthetic fungicides may be adulterated by unscrupulous traders and their incorrect use by illiterate farmers might result in poisoning and increase in pathogen resistance [105,106,107,108,109]. In the light of the highlighted challenges, there is a pressing need to search for alternative, less expensive/affordable, safer and environmentally friendly fungicides to control *Fusarium* pathogens and other pests in crop production. The search for applicable medicinal and aromatic plant species has attracted increasing attention in an effort towards the development of safer biopesticides.

## 3. The Potential of Natural Products from Medicinal Plants for Controlling *Fusarium* Pathogens

The control of pests using plant products was practiced as part of indigenous knowledge systems until technology took over and synthetic pesticides were developed and embraced quickly, because they were able to control many crop diseases successfully [110]. As a result, indigenous applications of plant products faded until researchers became aware of the harmful effect of synthetic pesticides on human health and the environment. Medicinal plant species have a long history of use by many ethnic groups for the treatment of various diseases in both humans and domestic animals [111,112]. Nevertheless, medicinal plant species have demonstrated the potential to be used as fungicides in the agricultural sector to protect crops against pathogens [112,113,114,115]. The idea behind the discovery of fungicides from plant species is based on their ability to synthesize diverse arrays of secondary metabolites or compounds, which function to defend the plant against microbes, insects and herbivores [116,117].

The use of plant products against fungal pathogens may inhibit the development of resistance due to the presence of different constituent antimicrobial compounds and their synergisms [118,119]. Products from medicinal plant species are arguably relatively safe, show low human toxicity and are eco-friendly [120]. They are easily biodegradable because natural products particularly from plants are inherently unstable with elevated temperatures and, consequently, they do not persist in the environment for a long time compared to conventional synthetic fungicides [80]. Nonetheless, it is important to evaluate the safety or toxicity and environmental fates of every alternative fungicide including biopesticides from medicinal plants. Biopesticides may produce residues and become toxic; hence, their maximum residue level in crops and animal products need to be established during the registration process [121]. Plant-based fungicides may be developed as products from the leaves or any part of the plant and used as essential oils, extracts or isolated compounds formulated into standardised products.

Reducing the use of conventional synthetic fungicides in the presence of effective natural products is a vital step towards sustainable crop production. In the following subsections, we review some studies conducted in the past 10 years on antifungal activity of plant extracts, essential oils and compounds isolated from plants against phytopathogenic *Fusarium* species.

### Plant Extracts, Essential Oils and Compounds with Antifungal Activity

Medicinal plant extracts have attracted attention in the pesticide industry as potential agents to control crop diseases in the field and during post-harvest storage. This is based on their antimicrobial properties due to spectrum of their constituent secondary metabolites such as phenols, polyphenols, flavonoids, glycosides, tannins, alkaloids and other compounds [122,123]. Table 2 shows the activity of extracts from some plant species evaluated for antifungal activity against phytopathogenic *Fusarium* species. Different solvent extracts obtained from 47 plant species belonging to 30 families were documented. The families with high frequencies of evaluated species against *Fusarium* pathogens were Solanaceae (with six species), followed by Combretaceae and Fabaceae (with four species each), and Euphorbiaceae (with three species). Plants in the Solanaceae family that were evaluated include *Nicotiana glauca*, *Solanum aculeastrum*, *Solanum mauritianum* and *Solanum seaforthianum*. Leaf extracts from these plants demonstrated potent in vitro activities (minimum inhibitory concentrations <1.0 mg/mL) against nine *Fusarium* species (Table 2). The *Solanum* species are regarded as invasive weeds, for which renewed biological control research has been advocated [124]. Their alternative use in the control of *Fusarium* pathogens could be beneficial for controlling their invasiveness. Extracts from species belonging to the Combretaceae and Fabaceae families similarly demonstrated potent activities against *Fusarium* species. While extracts could be prepared from different plant parts including roots, stems and leaves, most of the documented studies focused on leaf extracts. The use of leaves is particularly sustainable from a conservation point of view, as leaves are a renewable part that can be sustainably harvested without threatening plant growth and survival.

An important parameter to be considered is the choice of extraction solvents. In general, acetone, ethyl acetate, petroleum ether, chloroform, ethanol, methanol and water are commonly used for the extraction of various secondary metabolites from plants. Organic solvents such as acetone, ethyl acetate and petroleum ether demonstrated stronger antifungal activity against some *Fusarium* pathogens when compared to water extract obtained from the same plant species [125]. This observation correlated with the findings from several authors who reported that aqueous extract generally exhibited little or no antimicrobial activity compared to non-polar extracts [126,127,128]. This might be due to lower solubility of medicinal plant antifungal compounds in polar solvents as compared to non-polar solvents [129]. The polarity of constituent metabolites differs significantly and has influence on their solubility during extraction and thereafter in the antifungal activity of the extracts. On the other hand, the use of water extract would be applicable to resource-poor farmers since water is readily available; therefore, small-holder farmers can prepare crude plant extracts themselves. Bioactive water extracts are also particularly applicable in organic farming. Following the individual evaluation of plant extracts, a combination of bioactive plant extracts could result in stronger in vitro and in vivo antifungal activities due to possible synergistic antifungal activities of their constituent metabolites [130,131]. Solvents of different polarities may also be combined at varied ratios for improving extraction efficiency of bioactive constituents that may act synergistically. However, there remains a paucity of information on the combinational activity of plant extracts against plant pathogens as well as in vivo evaluation of bioactive extracts, which are important steps in developing plant-based biopesticides.

Several studies evaluated plant extracts against different *Fusarium* pathogens such as *F. verticilloides*, *F. proliferatum*, *F. oxysporum* and *F. solani*, all of which are known to infect cereals, fruits and vegetables. *Fusarium oxysporum* was the most frequently used pathogen (43 times) followed by *F. graminearum* and *F. verticilloides*, which were each used 23 times in the reported studies (Table 2). The least used pathogen was *F. semitectum*. Although the selection of *Fusarium* pathogen(s) for screening against plant extracts depends on many factors including the availability of pathogens and the target diseases to be controlled, the inclusion of multiple pathogenic strains in the screening process is more advantageous. The use of *Fusarium* pathogens with different morphological structures and defence mechanisms can help to discover active plant extracts against a wide spectrum of *Fusarium* pathogens. This approach could be beneficial for developing a biopesticide to manage different crop diseases caused by *Fusarium* pathogens.

The choice of assays used in evaluating medicinal plant extracts remains important to ensure the validity of extract potential. There are different screening methods or assays used to evaluate antifungal activity of plant extracts. The most common ones include microplate dilution and disk diffusion assays, with the microplate dilution assay being the most frequently used to evaluate antifungal activity of plant extracts against *Fusarium* pathogens (Table 2). The use of the agar diffusion method in determining antimicrobial activity of plant extracts is discouraged due to its pitfalls, including reproducibility issues between different laboratories and diffusion challenges with extracts of different polarities (particularly non-polar extracts) [132]. The measurement of the zone of inhibition depends on different factors such as the concentration and volume of test extracts, inoculum size and agar medium volume, amongst others, all of which make it difficult, if not impossible, to effectively compare antimicrobial activities reported as the inhibition zone of different extracts tested in different laboratories [132]. The use of an appropriate positive control is well known as a critical factor in validating antimicrobial assays [132]. Although other fungicides such as nystatin and ketoconazole may be used as a positive control, amphotericin B was used in most studies (Table 2). Of the studies consulted during the compilation of this review, at least 39 out of 51 studies included amphotericin B as a positive control. Compared to other fungicides, amphotericin B is easy to handle and store. Nevertheless, a number of studies evaluating the activity of plant extracts were conducted without including any positive control required to validate the experiment. In some other cases where a positive control was included in the experiments, the antifungal activity of the positive control was not reported. Antifungal activity studies without the use of any positive control raise validity concerns. The inclusion of the antifungal activity of standard positive controls can help to benchmark the antifungal potency of extracts and be used for inter-laboratory comparisons.

As presented in Table 2, the antifungal activity of plant extracts was expressed in terms of minimum inhibitory concentration (MIC), half-maximal inhibitory concentration (IC_50_) or percentage inhibition. Plant extract activities are usually dose dependent. Therefore, studies reporting percentage inhibition without specifying the concentration of the extract corresponding to such activity are of little value. Stating the antimicrobial activities of plant extracts in terms of their minimum inhibitory concentrations (MICs) is generally accepted as a minimum standard for reporting antimicrobial activity results [132]. Crude solvent extracts exhibiting MICs that are less than 1 mg/mL are generally regarded as having active/potential antimicrobial activity [133]. As shown in Table 2, extracts obtained from plant species such as *Milletia grandis*, *Solanum panduriforme* and *Ziziphus mucronata* demonstrated antifungal activity with a MIC value equal to or less than 0.01 mg/mL. Various extracts from *Combretum caffrum*, *C. erythrophyllum*, *C. molle*, *Harpephyllum caffrum*, *Lantana camara*, *Melia azedarach*, *Nicotiana glauca*, *Olea europaea*, *Passiflora suberosa*, *Quercus acutissima*, *Senna didymobotrya*, *Solanum aculeastrum*, *Solanum mauritianum*, *Vangueria infausta*, *Waburgia salutaris* and *Withania somnifera* demonstrated potent activities (with a MIC less than 1.0 mg/mL) against a number of *Fusarium* pathogens (Table 2). These plant extracts should be investigated further in vivo as part of efforts geared towards finding potential plant extracts to be developed into biopesticide products.

Few products developed from plants for application in crop protection are available on the market. Products such as Vertigo^®^ made from the seeds of *Cassia obtusifolia*, Milsana^®^ from *Reynoutria sachlinesis* and Owel^®^ made from an extract of *Macleaya cordata* are among good examples of natural products developed from botanicals and registered for application in crop protection [134,135]. Other botanical products available on the market for the treatment of plant diseases, particularly during post-harvest storage, include NeemPro^®^ and NeemAzal^®^. These products were reported to be successful as maize seed treatment agents [136,137]. The availability of such products indicates the possibility for formulating plant-based extracts against plant diseases caused by pathogenic *Fusarium* species.

Essential oils contain a mixture of different compounds such as monoterpenes, diterpenes, sesquiterpenes, aliphatic and other aromatic compounds that are volatile in nature [138,139,140]. Naturally, essential oils are usually obtained from medicinal plants, herbs, spices and aromatic plant species [141]. Different plant materials or parts including the flowers, leaves, barks, roots, seeds, fruits and whole plants can be utilized, depending on the plant species, for the extraction of essential oils [142,143]. They are commonly extracted by steam distillation or hydrodistillation process [144]. Essential oils are reputably used in traditional medicine, pharmaceutical, cosmetic and food industries [145,146]. Some oils are widely used as food preservatives, food flavours, appetizer promoters and perfumes [145,146].

The interest in the use of essential oils is due to their unique and excellent properties. Many studies have demonstrated antimicrobial activities, antioxidant activities, antiparasitic and insecticidal activities of essential oils [147,148,149,150,151]. Furthermore, essential oils have been investigated as control agents against growth of moulds and aflatoxin production [152,153,154,155]. Essential oils of some medicinal plant species were shown to be potential eco-friendly biocontrol agents [151,156]. These metabolites or substances can lead to new and different classes of botanical pesticides that may be used to control crop diseases including those caused by phytopathogenic *Fusarium* species. The application of essential oils against crop diseases is considered as a safe strategy to protect crops against pathogens. Because of their safety, the Federal Drug Administration (FDA) and Environmental Protection Agency (EPA) have allowed the use of certain essential oils in food [142,157]. Essential oils may be applicable in controlling post-harvest storage diseases. In addition to human safety, essential oils are fast or easily degraded in the environment and have low toxicity to non-target animals [158]. Thus, several studies have evaluated antifungal activity of essential oils obtained from different medicinal plant species against several *Fusarium* pathogens (Table 3). As indicated in Table 3, the essential oils from species belonging to the Lamiaceae, followed by the Apiaceae, Asteraceae and Myrtaceae plant families, were the most frequently evaluated against different *Fusarium* species. Essential oils from 26 species within the Lamiaceae family demonstrated various levels of activity against *Fusarium* pathogens. Essential oils from the genera *Origanum* and *Thymus* were the most utilized, followed by *Zataria multiflora*, *Melaleuca alternifolia* and *Cymbopogon citratus*. The very potent activities, based on the MIC values, reported in essential oils from *Artemissia sieberi* (MIC of 20 µg/mL against *F. solani*) and *Thymus kotschyanus* (MIC of 0.5 µg/mL against *F. oxysporum*) are particularly noteworthy. In Table 3, different methods used to evaluate activity of the essential oils were noted. Agar dilution, disc diffusion and microplate dilution methods were the most frequently used methods. The antifungal activity of essential oils was reported in a similar fashion as crude extracts (MIC values, IC_50_ values or percentage inhibition). Although there is no clear specified value used for classification to define whether an essential oil is highly active against *Fusarium* pathogens, the lower the MIC value, the higher the potency. The lack of a standardised assay method and reporting of results presents a challenge for effective comparison of the reported activities. Some of the assays were done without the use of appropriate controls, making it difficult to establish the validity of the assays used. Reporting of antifungal data without the use of positive control remains a challenge. About 40 experimental studies conducted to evaluate the activity of essential oils against *Fusarium* species were reported without a positive control (Table 3). Synthetic fungicide (fluconazole) was the mostly used positive control. Nonetheless, the recorded potent antifungal activity at low concentrations against some *Fusarium* species demonstrates the potential of developing biopesticides of plant origins. Further studies evaluating their in vivo potency against pathogenic *Fusarium* species are warranted. The plausible effectiveness of combining essential oils in developing suitable plant-based formulations merits scientific attention.

Medicinal plants are sources of bioactive secondary metabolites. These compounds belong to different chemical classes and have different structures. Of the plant families studied for the isolation of active compounds against *Fusarium* pathogens, Asteraceae was the most common, followed by Combretaceae and Zygophyllaceae. Compounds isolated from *Artemisia annua* were the most studied secondary metabolites against *Fusarium* pathogens (Table 4). These compounds were isolated from the leafy part of the plant. Bioactive compounds from medicinal plants are often present in very low amounts and may be difficult to purify on a large scale. However, they can be isolated, purified and characterized. The structures of isolated bioactive compounds may be used as a template during commercial production of biopesticides. Table 4 presents examples of isolated compounds from medicinal plants that demonstrated antifungal activity against several *Fusarium* pathogens. A number of isolated compounds showed strong potency (with minimum inhibitory concentration <20 µg/mL). Compounds isolated from medicinal plants are considered noteworthy when their reported minimum inhibitory concentration is less than 1 mg/mL [159]. Therefore, the isolated compounds reported in Table 4 demonstrated remarkable antifungal activity against a number of *Fusarium* pathogens.

**Table 2:** Medicinal plants evaluated for antifungal activity against *Fusarium* phytopathogenic species. The plant extracts were evaluated using different screening methods/assays, and their antifungal activities were reported in terms of minimum inhibitory concentration (MIC) or percentage inhibition values.

Plant Species (Family)	Solvents/Plant Parts Used	Method	Organism Tested	Positive Control	Activity of Positive Control	Results	References
*Aconitum laeve Royle* (Ranunculaceae)	Chloroform/tubers	poisoned food technique	*F. oxysporum*	Not stated	Not stated	Inhibition of 58.73 at 300 mg/mL	[160]
*Annona squamosa* L. (Annonaceae)	Methanol; Chloroform; Aqueous/leaf	broth dilution method	*F. solani*	100 mg/mL ketoconazole	Not stated	MIC value of 600; 300; 800 µg/mL	[161]
*Aristolochia elegans* Mast (Aristolochiaceae)	Acetone/leaf	serial microdilution assay	*F. oxysporum*	amphotericin B	7.5 µg/mL	MIC value of 0.08 mg/mL	[162,163]
*Artemisia absinthium* L. (Compositae)	Ethanol; Water/flowers	disk diffusion method	*F. oxysporum*	carbendazim	inhibition of 100% at 1% of the total volume	Inhibition of 65.69; 53.43 at 500 mg/L	[164]
	Ethanol; Water/leaf					Inhibition of 62.69; 51.33 at 500 mg/L	
	Ethyl acetate; Ethanol/roots					Inhibition of 72.45; 64.63 at 500 mg/L	
*Asparagus officinalis* L. (Asparagaceae)	Water	amended plate technique	*F. oxysporum*	Not stated	Not stated	Inhibition of 53.9 to 85.7	[165]
*Bauhinia galpinii* N.E.Br. (Fabaceae)	Acetone/leaf	microplate dilution method	*F. verticilloides*	amphotericin B	1.56 mg/mL	MIC value of 0.20 mg/mL	[166]
	Hot water; Methanol: Dichloromethane (1:1)/leaf	microplate dilution method	*F. graminearum*		0.004 mg/mL	MIC value of 0.30; 0.20 mg/mL	[167,168]
			*F. verticillioides*		0.006 mg/mL	MIC value of 3.13; 0.20 mg/mL	
			*F. oxysporum*		0.004 mg/mL	MIC value of 3.13; 1.56 mg/mL	
*Breonadia salicina* (Vahl) Hepper and J.R.I Wood (Rubiaceae)	Acetone; Hexane; Dichloromethane; Methanol/leaf	microplate method	*F. oxysporum*	amphotericin B	<0.02 mg/mL	MIC value of 0.32; 0.08; 0.16; 0.16 mg/mL	[115,169]
*Bucida buceras* L. (Combretaceae)	Acetone; Hexane; Dichloromethane; Methanol/leaf	microplate method	*F. oxysporum*	amphotericin B		MIC value of 0.02; 0.63; 0.32; 0.04 mg/mL	[115,169]
*Carpobrotus edulis* (L.) N.E.Br. (Aizoaceae)	Hot water; Methanol: Dichloromethane (1:1)/leaf	microplate dilution method	*F. graminearum*	amphotericin B	0.004 mg/mL	MIC value of 0.39; 3.13 mg/mL	[167,168]
			*F. verticillioides*		0.006 mg/mL	MIC value of 3.13; 0.10 mg/mL	
			*F. oxysporum*		0.004 mg/mL	MIC value of 3.13; 0.65 mg/mL	
*Chromolaena odorata* (L.) R.M.King & H.Rob. (Compositae)	Acetone/leaf	serial micro dilution assay	*F. oxysporum*	amphotericin B	7.5 µg/mL	MIC value of 0.08 mg/mL	[162,163]
*Combretum caffrum* (Eckl. & Zeyh.) Kuntze (Combretaceae)	Acetone/leaf	microplate dilution method	*F. verticilloides*	amphotericin B	1.56 mg/mL	MIC value of 0.31 mg/mL	[166]
*Combretum erythrophyllum* (Burch.) Sond. (Combretaceae)	Ethyl acetate; Acetone/leaf	microplate dilution method	*F. verticillioides*	amphotericin B	2.93 µg/mL	MIC value of 0.04; 0.04 mg/mL	[131]
	Water; Ethyl acetate; Acetone/leaf		*F. proliferetum*		0.37 µg/mL	MIC value of 0.31; 0.04; 0.04 mg/mL	
	Water; Ethyl acetate; Acetone/leaf		*F. solani*		0.37 µg/mL	MIC value of 0.16; 0.08; 0.04 mg/mL	
	Ethyl acetate; Acetone/leaf		*F. graminearum*		187.50 µg/mL	MIC value of 0.16; 0.08 mg/mL	
	Petroleum ether; Ethyl acetate; Acetone/leaf		*F. equisite*		187.50 µg/mL	MIC value of 0.04; 0.16; 0.04 mg/mL	[125]
	Petroleum ether; Ethyl acetate; Acetone/leaf		*F. oxysporum*		11.72 µg/mL	MIC value of 0.63; 0.31; 0.31 mg/mL	
	Water; Petroleum ether; Ethyl acetate; Acetone/leaf		*F. semitectum*		23.44 µg/mL	MIC value of 0.63; 0.63; 0.04; 0.04 mg/mL	
	Petroleum ether; Ethyl acetate; Acetone/leaf		*F. chlamydosporum*		23.44 µg/mL	MIC value of 0.04; 0.04; 0.08 mg/mL	
	Petroleum ether; Ethyl acetate; Acetone/leaf		*F. subglutinans*		93.75 µg/mL	MIC value of 0.04; 0.04; 0.08 mg/mL	
*Combretum molle* R. Br. ex G. Don (Combretaceae)	Ethyl acetate/leaf	microplate dilution method	*F. verticillioides*	amphotericin B	2.93 µg/mL	MIC value of 0.61 mg/mL	[131]
	Water; Ethyl acetate; Acetone/leaf		*F. proliferetum*		0.37 µg/mL	MIC value of 0.04; 0.04; 0.04 mg/mL	
	Water; Ethyl acetate; Acetone/leaf		*F. solani*		0.37 µg/mL	MIC value of 0.04; 0.04; 0.04 mg/mL	
	Ethyl acetate; Acetone/leaf		*F. graminearum*		187.50 µg/mL	MIC value of 0.63; 0.63 mg/mL	
	Water; Petroleum ether; Ethyl acetate; Acetone/leaf		*F. equisite*		187.50 µg/mL	MIC value of 0.63; 0.31; 0.16; 0.31 mg/mL	[125]
	Water; Petroleum ether; Ethyl acetate/leaf		*F. oxysporum*		11.72 µg/mL	MIC value of 0.31; 0.16; 0.16 mg/mL	
	Water; Petroleum ether; Ethyl acetate; Acetone/leaf		*F. semitectum*		23.44 µg/mL	MIC value of 0.63; 0.04; 0.08; 0.04 mg/mL	
	Water; Petroleum ether; Ethyl acetate/leaf		*F. chlamydosporum*		23.44 µg/mL	MIC value of 0.63; 0.04; 0.04 mg/mL	
	Water; Petroleum ether; Ethyl acetate; Acetone/leaf		*F. subglutinans*		93.75 µg/mL	MIC value of 0.63; 0.16; 0.63; 0.27 mg/ml	
	Acetone; Ethyl acetate; Dichloromethane/leaf	serial microplate dilution method	*F. oxysporum*		Not stated	MIC value of 0.19; 0.21; 0.16 mg/mL	[170]
*Euphorbia hirta* L. (Euphorbiaceae)	Water; Ethanol/leaf	agar plate dilution method	*F. oxysporum* *vasinfectum*	Not stated	Not stated	IC_50_ of 12.38 mg/mL; MIC value of 0.31 mg/mL and IC_50_ of 2.93 mg/mL	[171]
*Harpephyllum caffrum* Bernh. (Anacardiaceae)	Water; Ethyl acetate/leaf	microplate dilution method	*F. verticillioides*	amphotericin B	2.93 µg/mL	MIC value of 0.08; 0.08 mg/mL	[131]
	Water; Ethyl acetate; Acetone/leaf		*F. proliferetum*		0.37 µg/mL	MIC value of 0.04; 0.04; 0.04 mg/mL	
	Water; Ethyl acetate; Acetone/leaf		*F. solani*		0.37 µg/mL	MIC value of 0.08; 0.04; 0.63 mg/mL	
	Water; Ethyl acetate; Acetone/leaf		*F. graminearum*		187.50 µg/mL	MIC value of 0.16; 0.08; 0.31 mg/mL	
	Water; Petroleum ether; Ethyl acetate/leaf		*F. equisite*		187.50 µg/mL	MIC value of 0.31; 0.16; 0.16 mg/mL	[125]
	Water; Petroleum ether; Ethyl acetate/leaf		*F. oxysporum*		11.72 µg/mL	MIC value of 0.31; 0.16; 0.31 mg/mL	
	Water; Ethyl acetate/leaf		*F. chlamydosporum*		23.44 µg/mL	MIC value of 0.16; 0.16 mg/mL	
	Water; Petroleum ether; Ethyl acetate; Acetone/leaf		*F. subglutinans*		23.44 µg/mL	MIC value of 0.31; 0.08; 0.31; 0.78 mg/mL	
	Acetone/leaf	microplate dilution method	*F. verticilloides*		1.56 mg/mL	MIC value of 0.02 mg/mL	[166]
	Acetone; Hexane; Dichloromethane; Methanol/leaf	microplate method	*F. oxysporum*		<0.02 mg/mL	MIC value of 0.32; 0.16; 0.04; 0.39 mg/mL	[115,169]
	Hot water; Methanol: Dichloromethane (1:1)/leaf	microplate dilution method	*F. graminearum*		0.004 mg/mL	MIC value of 0.20; 0.78 mg/mL	[167,168]
			*F. verticillioides*		0.006 mg/mL	MIC value of 0.20; 0.39 mg/mL	
			*F. oxysporum*		0.004 mg/mL	MIC value of 0.52; 0.24 mg/mL	
*Ipomoea alba* L. (Convolvulaceae)	Acetone/leaf	serial micro dilution assay	*F. oxysporum*	amphotericin B	7.5 µg/mL	MIC value of 0.04 mg/mL	[162,163]
*Lantana camara* L. (Verbenaceae)	Water; Ethyl acetate; Acetone/leaf	microplate dilution method	*F. verticillioides*	amphotericin B	2.93 µg/mL	MIC value of 0.16; 0.16; 0.04 mg/mL	[131]
	Ethyl acetate; Acetone/leaf		*F. proliferetum*		0.37 µg/mL	MIC value of 0.04; 0.16 mg/mL	
	Ethyl acetate; Acetone/leaf		*F. solani*		0.37 µg/mL	MIC value of 0.04; 0.63 mg/mL	
	Water; Ethyl acetate; Acetone/leaf		*F. graminearum*		187.50 µg/mL	MIC value of 0.08; 0.63; 0.63 mg/mL	
	Water; Petroleum ether; Ethyl acetate/leaf		*F. equisite*		187.50 µg/mL	MIC value of 0.63; 0.31; 0.16 mg/mL	[125]
	Petroleum ether; Ethyl acetate/leaf		*F. oxysporum*		11.72 µg/mL	MIC value of 0.31; 0.63 mg/mL	
	Petroleum ether; Ethyl acetate/leaf		*F. semitectum*		23.44 µg/mL	MIC value of 0.08; 0.04 mg/mL	
	Water; Acetone/leaf		*F. chlamydosporum*		23.44 µg/mL	MIC value of 0.16; 0.16 mg/mL	
	Water; Petroleum ether; Ethyl acetate; Acetone/leaf		*F. subglutinans*		93.75 µg/mL	MIC value of 0.04; 0.04; 0.04; 0.39 mg/mL	
*Maesa lanceolata* Forsk (Primulaceae)	Hot water: Methanol: Dichloromethane (1:1)/leaf	microplate dilution method	*F. graminearum*	amphotericin B	0.004 mg/mL	MIC value of 0.20; 0.78 mg/mL	[167,168]
			*F. verticillioides*		0.006 mg/mL	MIC value of 0.20; 0.78 mg/mL	
			*F. oxysporum*		0.004 mg/mL	MIC value of 0.26; 0.08 mg/mL	
*Markhamia obtusifolia* (Baker) Sprague (Bignoniaceae)	Acetone/leaf	microplate dilution method	*F. verticilloides*	amphotericin B	1.56 mg/mL	MIC value of 0.31 mg/mL	[166]
*Melia azedarach* L. (Meliaceae)	Water; Ethyl acetate/leaf	microplate dilution method	*F. verticillioides*	amphotericin B	2.93 µg/mL	MIC value of 0.16; 0.08 mg/mL	[131]
	Water; Ethyl acetate/leaf		*F. proliferetum*		0.37 µg/mL	MIC value of 0.04; 0.08 mg/mL	
	Water; Ethyl acetate; Acetone/leaf		*F. solani*		0.37 µg/mL	MIC value of 0.08; 0.04; 0.63 mg/mL	
	Water; Ethyl acetate; Acetone/leaf		*F. graminearum*		187.50 µg/mL	MIC value of 0.08; 0.16; 0.63 mg/mL	
	Water; Petroleum ether; Ethyl acetate/leaf		*F. equisite*		187.50 µg/mL	MIC value of 0.31; 0.16; 0.16 mg/mL	[125]
	Water; Petroleum ether; Ethyl acetate/leaf		*F. oxysporum*		11.72 µg/mL	MIC value of 0.16; 0.08; 0.16 mg/mL	
	Petroleum ether; Ethyl acetate/leaf		*F. semitectum*		23.44 µg/mL	MIC value of 0.31; 0.63 mg/mL	
	Water; Petroleum ether; Ethyl acetate; Acetone/leaf		*F. chlamydosporum*		23.44 µg/mL	MIC value of 0.31; 0.63; 0.04; 0.08 mg/mL	
	Water; Petroleum ether; Ethyl acetate; Acetone/leaf		*F. subglutinans*		93.75 µg/mL	MIC value of 0.16; 0.16; 0.08; 0.63 mg/mL	
*Melianthus comosus* Vahl. (Melianthaceae)	Carbon tetrachloride; Diethyl ether; Dichloromethane; Chloroform; Acetone; Ethanol; Ethyl acetate/leaf	serial microdilution assay	*F. oxysporum*	Not stated	Not stated	MIC value of 0.63; 0.63; 0.16; 0.16; 0.04; 0.08; 0.78 mg/mL	[172,173]
*Milletia grandis* (E. Mey) Skeels (Fabaceae)	Hot water; Methanol: Dichloromethane (1:1)/leaf	microplate dilution method	*F. graminearum*	amphotericin B	0.004 mg/mL	MIC value of 0.01; 0.78; mg/mL	[167,168]
			*F. verticillioides*		0.006 mg/mL	MIC value of 0.10; 0.65 mg/mL	
			*F. oxysporum*		0.004 mg/mL	MIC value of 0.01; 0.01 mg/mL	
	Methanol: Dichloromethane (1:1)/leaf	Not stated	*F. graminarium*	Not stated	Not stated	MIC value of 0.01 mg/mL	[174]
		Not stated	*F. oxysporum*	Not stated	Not stated	MIC value of 0.39 mg/mL	[174]
*Momordica charantia* Linn. (Cucurbitaceae)	Seed	Not stated	*F. solani*	Not stated	Not stated	MIC value of 0.08 mg/mL and Inhibition of 57.216 at 125 µg/mL	[175,176]
*Mystroxylon aethiopicum* (Thunb.) Loes (Celastraceae)	Acetone/leaf	microplate dilution method	*F. verticilloides*	amphotericin B	1.56 mg/mL	MIC value of 0.16 mg/mL	[166]
*Nicotiana glauca* Graham (Solanaceae)	Water; Ethyl acetate/leaf	microplate dilution method	*F. verticillioides*	amphotericin B	2.93 µg/mL	MIC value of 0.04; 0.16 mg/mL	[131]
	Water; Ethyl acetate/leaf		*F. proliferetum*		0.37 µg/mL	MIC value of 0.04; 0.04 mg/mL	
	Water; Ethyl acetate; Acetone/leaf		*F. solani*		0.37 µg/mL	MIC value of 0.16; 0.08; 0.63 mg/mL	
	Water; Ethyl acetate; Acetone/leaf		*F. graminearum*		187.50 µg/mL	MIC value of 0.16; 0.16; 0.08 mg/mL	
*Olea europaea* L. (Oleaceae)	Water; Ethyl acetate; Acetone/leaf	microplate dilution method	*F. verticillioides*	amphotericin B	2.93 µg/mL	MIC value of 0.16; 0.16; 0.04 mg/mL	[131]
	Water; Ethyl acetate/leaf		*F. proliferetum*		0.37 µg/mL	MIC value of 0.04; 0.04 mg/mL	
	Water; Ethyl acetate/leaf		*F. solani*		0.37 µg/mL	MIC value of 0.04; 0.04 mg/mL	
	Water; Ethyl acetate; Acetone/leaf		*F. graminearum*		187.50 µg/mL	MIC value of 0.02; 0.02; 0.63 mg/mL	
	Petroleum ether; Ethyl acetate/leaf		*F. equisite*		187.50 µg/mL	MIC value of 0.31; 0.31 mg/mL	[125]
	Water; Petroleum ether; Ethyl acetate/leaf		*F. oxysporum*		11.72 µg/mL	MIC value of 0.63; 0.31; 0.31 mg/mL	
	Acetone/leaf		*F. semitectum*		23.44 µg/mL	MIC value of 0.04 mg/mL	
	Water; Acetone/leaf		*F. chlamydosporum*		23.44 µg/mL	MIC value of 0.04; 0.3l mg/mL	
	Water; Petroleum ether; Ethyl acetate; Acetone/leaf		*F. subglutinans*		93.75 µg/mL	MIC value of 0.31; 0.31; 0.31; 0.08 mg/mL	
*Olinia ventosa* (L.) Cufod (Penaeaceae)	Acetone; Hexane; Dichloromethane; Methanol/leaf	microplate method	*F. oxysporum*	amphotericin B	<0.02 mg/mL	MIC value of 0.63; 0.31; 0.16; 0.16 mg/mL	[115,169]
*Passiflora suberosa* L. (Passifloraceae)	Acetone/leaf	serial microdilution assay	*F. oxysporum*	amphotericin B	7.5 μg/mL	MIC value of 0.04 mg/mL	[162,163]
*Quercus acutissima* Carruth. (Fagaceae)	Water; Ethyl acetate/leaf	Microplate dilution method	*F. verticillioides*	amphotericin B	2.93 µg/mL	MIC value of 0.08; 0.08 mg/mL	[131]
	Water; Ethyl acetate/leaf		*F. proliferetum*		0.37 µg/mL	MIC value of 0.04; 0.04 mg/mL	
	Water; Ethyl acetate; Acetone/leaf		*F. solani*		0.37 µg/mL	MIC value of 0.04; 0.04; 0.31 mg/mL	
	Water; Ethyl acetate/leaf		*F. graminearum*		187.50 µg/mL	MIC value of 0.02; 0.02 mg/mL	
	Water; Petroleum ether; Ethyl acetate/leaf		*F. equisite*		187.50 µg/mL	MIC value of 0.31; 0.16; 0.08 mg/mL	[125]
	Water; Petroleum ether; Ethyl acetate/leaf		*F. oxysporum*		11.72 µg/mL	MIC value of 0.16; 0.08; 0.16 mg/mL	
	Water; Petroleum ether; Ethyl acetate; Acetone/leaf		*F. semitectum*		23.44 µg/mL	MIC value of 0.63; 0.31; 0.31; 0.16 mg/mL	
	Water; Petroleum ether; Ethyl acetate/leaf		*F. chlamydosporum*		23.44 µg/mL	MIC value of 0.04; 0.16; 0.04 mg/mL	
	Water; Petroleum ether; Ethyl acetate/leaf		*F. subglutinans*		93.75 µg/mL	MIC value of 0.16; 0.08; 0.63 mg/mL	
*Rhus muelleri* Standl. & F.A.Barkley (Anacardiaceae)	Ethanol/leaf	agar dilution method	*F. oxysporum f. sp. lycopersici*	Not stated	Not stated	MIC value of 0.39 mg/mL and inhibition of 56.8% at 4500 ppm	[177,178]
*Ricinus communis* L (Euphorbiaceae)	Acetone/leaf	microplate dilution method	*F. verticilloides*	amphotericin B	1.56 mg/mL	MIC value of 0.39 mg/mL	[166]
	Hot water/leaf		*F. graminearum*		0.004 mg/mL	MIC value of 0.20 mg/mL	[167,168]
	Hot water; Methanol: Dichloromethane (1:1)/leaf		*F. verticillioides*		0.006 mg/mL	MIC value of 0.02; 0.78 mg/mL	
	Hot water/leaf		*F. oxysporum*		0.004 mg/mL	MIC value of 0.16 mg/mL	
*Rumex vesicarius* L. (Polygonaceae)	Aqueous extract or Water/shoot	agar dilution method	*F. oxysporum*	Not stated	Not stated	MIC value of 0.625 mg/mL and Inhibition of 50.97 at 25 mg/mL	[179,180]
*Salacia macrosperma* *Wight*. (Celastraceae)	Ethyl acetate; Methanol/leaf	disc diffusion	*F. moniliforme*	nystatin	0.078 mg/mL	MIC value of 0.312; 0.312 mg/mL	[181]
	Methanol/leaf		*F. oxysporum*		0.156 mg/mL	MIC value of 0.625 mg/mL	
*Schotia brachypetala* Sond. (Fabaceae)	Water; Ethyl acetate/leaf	microplate dilution method	*F. verticillioides*	amphotericin B	2.93 µg/mL	MIC value of 0.31; 0.16 mg/mL	[131]
	Water; Ethyl acetate/leaf		*F. proliferetum*		0.37 µg/mL	MIC value of 0.04; 0.04 mg/mL	
	Ethyl acetate; Acetone/leaf		*F. solani*		0.37 µg/mL	MIC value of 0.63; 0.04 mg/mL	
	Water; Ethyl acetate; Acetone/leaf		*F. graminearum*		187.50 µg/mL	MIC value of 0.16; 0.16; 0.31 mg/mL	
*Senna didymobotrya* (Fresen.) H.S. Irwin & Barneby (Fabaceae)	Water; Ethyl acetate; Acetone/leaf	microplate dilution method	*F. verticillioides*	amphotericin B	2.93 µg/mL	MIC value of 0.16; 0.08; 0.04 mg/mL	[131]
	Ethyl acetate/leaf	microplate dilution method	*F. proliferetum*		0.37 µg/mL	MIC value of 0.04 mg/mL	
	Water; Ethyl acetate; Acetone/leaf		*F. solani*		0.37 µg/mL	MIC value of 0.08; 0.08; 0.63 mg/mL	
	Water; Ethyl acetate; Acetone/leaf		*F. graminearum*		187.50 µg/mL	MIC value of 0.16; 0.63; 0.16 mg/mL	
	Water; Petroleum ether; Ethyl acetate/leaf		*F. equisite*		187.50 µg/mL	MIC value of 0.16; 0.31; 0.31 mg/mL	[125]
	Water; Petroleum ether; Ethyl acetate/leaf		*F. oxysporum*		11.72 µg/mL	MIC value of 0.31; 0.16; 0.16 mg/mL	
	Water; Acetone/leaf		*F. chlamydosporum*		23.44 µg/mL	MIC value of 0.63; 0.04 mg/mL	
	Water; Petroleum ether; Ethyl acetate; Acetone/leaf		*F. subglutinans*		23.44 µg/mL	MIC value of 0.08; 0.04; 0.08; 0.26 mg/mL	
*Solanum aculeastrum* Dunal (Solanaceae)	Acetone/leaf	microplate dilution method	*F. verticilloides*	amphotericin B	1.56 mg/mL	MIC value of 0.39 mg/mL	[166]
	Hot water; Methanol: Dichloromethane (1:1)/leaf	microplate dilution method	*F. graminearum*		0.004 mg/mL	MIC value of 0.78; 0.39 mg/mL	
			*F. verticillioides*		0.006 mg/mL	MIC value of 0.40; 0.20 mg/mL	
	Hot water/leaf		*F. oxysporum*		0.004 mg/mL	MIC value of 0.78 mg/mL	
*Solanum mauritianum* Scop. (Solanaceae)	Water; Ethyl acetate/leaf	microplate dilution method	*F. verticillioides*	amphotericin B	2.93 µg/mL	MIC value of 0.04; 0.16 mg/mL	[131]
	Water; Ethyl acetate/leaf		*F. proliferetum*		0.37 µg/mL	MIC value of 0.04; 0.04 mg/mL	
	Water; Ethyl acetate; Acetone/leaf		*F. solani*		0.37 µg/mL	MIC value of 0.04; 0.04; 0.63 mg/mL	
	Water; Ethyl acetate; Acetone/leaf		*F. graminearum*		187.50 µg/mL	MIC value of 0.16; 0.04; 0.16 mg/mL	
	Water; Petroleum ether; Ethyl acetate/leaf		*F. equisite*		187.50 µg/mL	MIC value of 0.31; 0.08; 0.31 mg/mL	[125]
	Water; Petroleum ether; Ethyl acetate/leaf		*F. oxysporum*		11.72 µg/mL	MIC value of 0.31; 0.08; 0.04 mg/mL	
	Water/leaf		*F. semitectum*		23.44 µg/mL	MIC value of 0.63 mg/mL	
	Water; Petroleum ether; Ethyl acetate; Acetone/leaf		*F. chlamydosporum*		23.44 µg/mL	MIC value of 0.31; 0.31; 0.31; 0.08 mg/mL	
	Water; Petroleum ether; Ethyl acetate/leaf		*F. subglutinans*		93.75 µg/mL	MIC value of 0.16; 0.04; 0.04 mg/mL	
*Solanum panduriforme* E. Mey. (Solanaceae)	Hot water; Methanol: Dichloromethane (1:1)/leaf	microplate dilution method	*F. graminearum*	amphotericin B	0.004 mg/mL	MIC value of 0.10; 0.78 mg/mL	[167,168]
			*F. verticillioides*		0.006 mg/mL	MIC value of 0.20; 0.39 mg/mL	
			*F. oxysporum*		0.004 mg/mL	MIC value of 0.01; 0.08 mg/mL	
*Solanum seaforthianum* Andrews (Solanaceae)	Acetone/leaf	serial microdilution assay	*F. oxysporum*	amphotericin B	7.5 μg/mL	MIC value of 0.31 mg/mL	[162,163]
*Spirostachys africana* Sond. (Euphorbiaceae)	Acetone/leaf	microplate dilution method	*F. verticilloides*	amphotericin B	1.56 mg/mL	MIC value of 0.63 mg/mL	[166]
*Strychnos mitis* S.Moore (Loganiaceae)	Acetone/leaf	microplate dilution method	*F. verticilloides*	amphotericin B	1.56 mg/mL	MIC value of 0.24 mg/mL	[166]
*Vangueria infausta* Burch (Rubiaceae)	Water; Ethyl acetate/leaf	microplate dilution method	*F. verticillioides*	amphotericin B	2.93 µg/mL	MIC value of 0.08; 0.04 mg/mL	[131]
	Water; Ethyl acetate; Acetone/leaf		*F. proliferetum*		0.37 µg/mL	MIC value of 0.04; 0.04; 0.63 mg/mL	
	Water; Ethyl acetate; Acetone/leaf		*F. solani*		0.37 µg/mL	MIC value of 0.04; 0.04; 0.31 mg/mL	
	Water; Ethyl acetate; Acetone/leaf		*F. graminearum*		187.50 µg/mL	MIC value of 0.31; 0.16; 0.32 mg/mL	
	Acetone; Hexane; Dichloromethane/leaf		*F. oxysporum*		< 0.02 mg/mL	MIC value of 0.63; 0.32; 0.32 mg/mL	[115,169]
*Vangueria infausta* Burch (Rubiaceae)	Water; Petroleum ether; Ethyl acetate; Acetone/leaf	microplate dilution method	*F. equisite*	amphotericin B	187.50 µg/mL	MIC value of 0.63; 0.31; 0.16; 0.63 mg/mL	[125]
	Water; Petroleum ether; Ethyl acetate/leaf		*F. oxysporum*		11.72 µg/mL	MIC value of 0.31; 0.16; 0.16 mg/mL	
	Water; Petroleum ether; Ethyl acetate; Acetone/leaf		*F. semitectum*		23.44 µg/mL	MIC value of 0.63; 0.08; 0.16; 0.04 mg/mL	
	Water; Petroleum ether; Ethyl acetate; Acetone/leaf		*F. chlamydosporum*		23.44 µg/mL	MIC value of 0.63; 0.31; 0.08; 0.16 mg/mL	
	Water; Petroleum ether; Ethyl acetate; Acetone/leaf		*F. subglutinans*		93.75 µg/mL	MIC value of 0.31; 0.31; 0.31; 0.78 mg/mL	
*Warburgia salutaris* (G. Bertol) Chiov. (Canellaceae)	Hot water/leaf	microplate dilution method	*F. graminearum*	amphotericin B	0.004 mg/mL	MIC value of 0.10 mg/mL	[167,168]
	Hot water; Methanol: Dichloromethane (1:1)/leaf		*F. verticillioides*		0.006 mg/mL	MIC value of 0.10; 0.78 mg/mL	
			*F. oxysporum*		0.004 mg/mL	MIC value of 0.10; 0.10 mg/mL	
	Acetone/leaf		*F. verticilloides*		1.56 mg/mL	MIC value of 0.63 mg/mL	[166]
*Withania somnifera* (L.) Dunal (Solanaceae)	Water; Ethyl acetate; Acetone/leaf	microplate dilution method	*F. verticillioides*	amphotericin B	2.93 µg/mL	MIC value of 0.08; 0.08; 0.04 mg/mL	[131]
	Water; Ethyl acetate; Acetone/leaf		*F. proliferetum*		0.37 µg/mL	MIC value of 0.04; 0.04; 0.63 mg/mL	
	Water; Ethyl acetate/leaf		*F. solani*		0.37 µg/mL	MIC value of 0.08; 0.04 mg/mL	
	Water; Petroleum ether; Ethyl acetate/leaf		*F. equisite*		187.50 µg/mL	MIC value of 0.63; 0.16; 0.31 mg/mL	[125]
	Water; Petroleum ether; Ethyl acetate/leaf		*F. oxysporum*		11.72 µg/mL	MIC value of 0.16; 0.08; 0.08 mg/mL	
	Water; Petroleum ether; Ethyl acetate/leaf		*F. semitectum*		23.44 µg/mL	MIC value of 0.63; 0.04; 0.08 mg/mL	
	Water; Ethyl acetate; Acetone/leaf		*F. chlamydosporum*		23.44 µg/mL	MIC value of 0.63; 0.63; 0.16 mg/mL	
	Water; Petroleum ether; Ethyl acetate; Acetone/leaf		*F. subglutinans*		93.75 µg/mL	MIC value of 0.08; 0.63; 0.31; 0.63 mg/mL	
*Xylotheca kraussiana* Hochst. (Achariaceae)	Acetone/leaf	microplate dilution method	*F. verticilloides*	amphotericin B	1.56 mg/mL	MIC value of 0.63 mg/mL	[166]
	Acetone; Hexane; Dichloromethane/leaf		*F. oxysporum*		<0.02 mg/mL	MIC value of 0.32; 0.32; 0.32 mg/mL	[115,169]
	Methanol/leaf		*F. oxysporum*			MIC value of 0.08 mg/mL	
*Ziziphus mucronata* Wild. (Rhamnaceae)	Hot water; Methanol: Dichloromethane (1:1)/leaf	microplate dilution method	*F. graminearum*	amphotericin B	0.006 mg/mL	MIC value of 0.01; 0.78 mg/mL	[167,168]
			*F. oxysporum*		0.004 mg/mL	MIC value of 0.39; 0.39 mg/mL	[167,168]

**Table 3:** Antifungal activity of essential oils obtained from plants used in traditional medicine. The oil samples were evaluated against *Fusarium* phytopathogenic species using different methods and their activities were reported as minimum inhibitory concentration, half-maximal inhibitory concentration (IC_50_) or percentage inhibition values.

Plant Species (Family) Source of Essential Oil	Method	Organism Tested	Positive Control	Activity of Positive Control	Results	Reference
*Achillea biebersteinii* Afan. ex Hub.-Mor. (Asteraceae)	disc diffusion method	*F. verticilloides*	Not stated	Not stated	Inhibition of 92.9% at 25 µL	[182]
*Aconitum laeve* Royle (Ranunculaceae)	disc diffusion method	*F. oxysporum*	amphotericin B; clotrimazole	200; 300 µg/mL	MIC value of 300 µg/mL	[157]
*Aloysia polystachya* (Griseb.) Moldenke Biurrum 8755 (Verbenaceae)	disc diffusion method	*F. verticillioides*	Not stated	Not stated	IC_50_ of 1082.43 µg/mL	[158]
*Artemisia sieberi* Besser. (Asteraceae)	broth microdilution method	*F. solani*	Itraconazole; Fluconazole; Ketoconazole	7; 18; 12 µg/mL	MIC value of 20 µg/mL	[183]
		*F. oxysporum*		9; 10; 9 µg/mL	MIC value of 60 µg/mL	
*Asarum heterotropoides* var. mandshuricum (Aristolochiaceae)	disc diffusion method	*F. avenaceum*	nystatin	Not stated	MIC_50_ of 0.61 mg/mL	[184]
		*F. trichothecioides*			MIC_50_ of 0.72 mg/mL	
		*F. sporotrioides*			MIC_50_ of 0.83 mg/mL	
*Bupleurum falcatum* L. (Apiaceae)	broth microdilution method	*F. oxysporum*	amphotericin B	0.5 µg/mL	MIC of 2 µg/mL	[185]
*Chenopodium ambrosioides* L. (Chenopodiaceae)	disc diffusion method	*F. verticillioides*	Not stated	Not stated	IC_50_ of 243.12 µg/mL	[158]
*Cannabis sativa* L. (Cannabidaceae)	agar dilution method	*F. oxysporum*	Not stated	Not stated	Inhibition of 93.58% at 1 µL/mL	[155]
		*F. verticillioides*			Inhibition of 88.17% at 1 µL/mL	
*Cinnamomum camphora* (Lauraceae)	toxic medium assay	*F. oxysporum isolate* S-1187.	ICA-Thiabendazole^®^ 500SC	Not stated	Inhibition of 49% at 3000 µL/L	[186]
*Cinnamon zeylanicum* (Lauraceae)		*F. oxysporum isolate* S-1187.			Inhibition of 92% at 500 µL/L	
*Citrus aurantium* (Rutaceae)	agar dilution method.	*F. oxysporum*	Not stated	Not stated	Inhibition of 57.75% at 1 µL/mL	[155]
		*F. verticillioides*			Inhibition of 57.40% at 1 µL/mL	
*Citrus reticulata* L. (Rutaceae)	poisoned food technique	*F. oxysporum*	Not stated	Not stated	Inhibition of 70% at 0.15 mL/100 mL	[187]
*Citrus sinensis* L. (Rutaceae)	disc diffusion method	*F. verticillioides*	Not stated	Not stated	IC_50_ of 1604.82 µL/L	[158]
*Coriandrum sativum* L. (Apiaceae)	microdilution technique	*F. solani*	fluconazole	Not stated	MIC value of 0.97 mg/mL	[188]
*Cuminum cyminum* (Apiaceae)	broth dilution method	*F. solani isolates*	Not stated	Not stated	MIC value of 69 µg/mL	[189]
		*F. oxysporum isolates*	Not stated	Not stated	MIC value of 72 µg/mL	[189]
		*F. verticillioides isolates*			MIC value of 73 µg/mL	
		*F. poae isolates*			MIC value of 130 µg/mL	
		*F. equiseti isolates*			MIC value of 75 µg/mL	
*Curcuma longa* L. (Zingiberaceae)	microwell dilution method	*F. graminearum*	Nystatin; Amphotericin B	2200; 1400 µg/mL	MIC value of 2450 µg/mL	[190]
*Cymbopogon citratus*, Stapf. (Poaceae)	toxic medium assay	*F. oxysporum isolate* S-1187.	ICA-Thiabendazole^®^ 500SC	Not stated	Inhibition of 100% at 2500 µL/L	[186]
*Cymbopogon nardus* (L.) Rendle (Poaceae)	agar dilution method	*F. oxysporum*	Not stated	Not stated	Inhibition of 85.56% at 1 µL/mL	[155]
		*F. verticillioides*			Inhibition of 75.74% at 1 µL/mL	
*Daucus carota* L. var. Chantenay (Apiaceae)	agar dilution method	*F. verticillioides*	Not stated	Not stated	Inhibition of 56.80% at 1 µL/mL	[155]
*Echinophora platyloba* DC. (Apiaceae)	agar dilution and disk diffusion methods	*F. oxysporum*	Not stated	Not stated	Inhibition of 51.8% at 1 µL/L	[191]
*Eucalyptus* sp. (Myrtaceae)	disk diffusion method	*F*. *graminearum*	Not stated	Not stated	Inhibition of 56% at 1000 µL/L	[192]
		*F*. *asiaticum*			Inhibition of 67% at 1500 µL/L	
		*F*. *redolens f. sp. dianthus*			Inhibition of 55.11% at 1000 µL/L	
		*F*. *verticillioides*			Inhibition of 72.44% at 1500 µL/L	
		*F*. *oxysporum f. sp. lentis*			Inhibition of 55.11% at 1500 µL/L	
*Foeniculum vulgare* Mill. (Apiaceae)	broth dilution method	*F. solani isolates*	Not stated	Not stated	MIC value of 77 µg/mL	[189]
		*F. oxysporum isolates*			MIC value of 72 µg/mL	
		*F. verticillioides isolates*			MIC value of 77 µg/mL	
		*F. poae isolates*			MIC value of 96 µg/mL	
		*F. equiseti isolates*			MIC value of 63 µg/mL	
*Foeniculum vulgare* Mill. (Apiaceae) *fruits*	agar disk diffusion	*F. fujikuroi*	Not stated	Not stated	MIC value of 2.0 µL/mL	[193]
*Helichrysum splendidum* (Thunb.) Less. (Asteraceae)	toxic medium assay	*F. oxysporum isolate* S-1187.	ICA-Thiabendazole^®^ 500SC	Not stated	Inhibition of 58% at 3000 µL/L	[186]
*Heracleum persicum* Desf. Ex Fischer. (Apiaceae)	broth dilution method	*F. solani isolates*	Not stated	Not stated	MIC value of 675 µg/mL	[189]
		*F. oxysporum isolates*	Not stated	Not stated	MIC value of 70 µg/mL	[189]
		*F. verticillioides isolates*			MIC value of 225 µg/mL	
		*F. poae isolates*			MIC value of 952 µg/mL	
		*F. equiseti isolates*			MIC value of 1062 µg/mL	
		*F. solani*	Itraconazole; Fluconazole; Ketoconazole	7; 18; 12 µg/mL	MIC value of 480 µg/mL	[183]
		*F. oxysporum*		9; 10; 9 µg/mL	MIC value of 530 µg/mL	
*Illicium verum* Hook.f. (Schisandraceae)	microdilution technique	*F. solani*	fluconazole	Not stated	MIC value of 0.93 mg/mL	[188]
		*F. verticillioides*			MIC value of 0.70 mg/mL	
*Laurus nobilis* L. (Lauraceae)	disc diffusion method	*F. verticillioides*	Not stated	Not stated	IC_50_ of 1846.87 µL/L	[158]
*Lavandula angustifolia* Mill. (Lamiaceae)	agar dilution method	*F. verticillioides*	Not stated	Not stated	Inhibition of 68.64% at 1 µL/mL	[155]
*Cymbopogon citratus*, mycorrhizal lemongrass. (Poaceae)	food poisoning method	*F. solani*	Ridomil plus 44 WP	100% at 250 ppm	Inhibition of 89% at 250 ppm	[194]
*Cymbopogon citratus*, non-mycorrhizal lemongrass. (Poaceae)					Inhibition of 71% at 250 ppm	
*Lippia rehmannii* H.Pearson (Verbenaceae)	toxic medium assay	*F. oxysporum isolate* S-1187.	ICA-Thiabendazole^®^ 500SC	Not stated	Inhibition of 72% at 500 µL/L	[186]
*Lippia scaberrima* Sond. (Verbenaceae)					Inhibition of 87% at 3000 µL/L	
*Matricaria recutita* (L.) syn. (Asteraceae)	microbioassay technique	*F. oxysporum*	ketoconazole	29.7% at 10 mg/disk	Inhibition of 56.0% at 62.5 µg/mL	[195]
*Melaleuca alternifolia* (Myrtaceae)	microdilution technique	*F. verticillioides*	fluconazole	Not stated	MIC value of 0.86 mg/mL	[188]
		F. oxysporum			MIC value of 0.91 mg/mL	
*Melaleuca alternifolia* L. (Maiden and Betche) Cheel. (Myrtacea)	agar dilution method	*F. oxysporum*	Not stated	Not stated	Inhibition of 58.29% at 1 µL/mL	[155]
		*F. verticillioides*			Inhibition of 56.80% at 1 µL/mL	
*Mentha spicata* L. (spearmint) (Lamiaceae)	toxic medium assay	*F. oxysporum isolate* S-1187.	ICA-Thiabendazole^®^ 500SC	Not stated	Inhibition of 79% at 2000 µL/L	[186]
*Minthostachys verticillata* Griseb. (Lamiaceae)	disc diffusion method	*F. verticillioides*	Not stated	Not stated	IC_50_ of 1552.43 µL/L	[158]
*Myrcia ovata* Cambesse (Myrtaceae)	contact	*F. solani*	Viper 700 (0.07% *w*/*v*)	Not stated	Inhibition of 53.9% at 100 µL/mL	[54]
*Nepeta cataria* L. (Lamiaceae)	agar dilution method,	*F. verticillioides*	Not stated	Not stated	Inhibition of 91.72% at 1µL/mL	[155]
		*F. oxysporum*			Inhibition of 97.86% at 1 µL/mL	
*Ocimum basilicum* L. (Lamiaceae)	agar dilution method.	*F. oxysporum*	Not stated	Not stated	Inhibition of 74.87% at 1 µL/mL	[155]
		*F. verticillioides*			Inhibition of 77.51% at 1 µL/mL	
*Origanum heracleoticum* L. (Lamiaceae)	microdilution technique	*F. solani*	fluconazole	Not stated	MIC value of 0.14 mg/mL	[188]
		*F. tricinctum*			MIC value of 0.14 mg/mL	
		*F. sporotrichioides*			MIC value of 0.28 mg/mL	
		*F. verticillioides*			MIC value of 0.14 mg/mL	
		*F. oxysporum*			MIC value of 0.07 mg/mL	
		*F. semitectum*			MIC value of 0.28 mg/mL	
		*F. equiseti*			MIC value of 0.28 mg/mL	
*Origanum majorana* L. (Lamiaceae)	agar dilution method	*F. oxysporum*	Not stated	Not stated	Inhibition of 59.36% at 1 µL/mL	[155]
		*F. verticillioides*			Inhibition of 75.74% at 1 µL/mL	
*Origanum vulgare* L. (Lamiaceae)	broth microdilution method	*F. solani*	Itraconazole; Fluconazole; Ketoconazole	7; 18; 12 µg/mL	MIC value of 50 µg/mL	[183]
		*F. oxysporum*		9; 10; 9 µg/mL	MIC value of 50 µg/mL	
*Origanum vulgare* L. spp. virens (Lamiaceae)	disc diffusion method	*F. verticillioides*	Not stated	Not stated	IC_50_ of 101.71 µL/L	[158]
*Origanum vulgare* L. spp. vulgare (Lamiaceae)		*F. verticillioides*			IC_50_ of 108.27 µL/L	
*Origanum x applii* (Domin Boros) (Lamiaceae)	disc diffusion method	*F. verticillioides*	Not stated	Not stated	IC_50_ of 66.79 µL/L	[158]
*Pelargonium graveolens* L’Heritier. (Geraniaceae)	microdilution technique	*F. equiseti*	fluconazole	Not stated	MIC value of 0.66 mg/mL	[188]
*Pelargonium odoratissimum* (Geraniaceae)	agar dilution method	*F. culmorum*	Not stated	Not stated	Inhibition of 65.45% at 1 µL/L	[196]
*Pelargonium roseum* L. (Geraniaceae)	agar dilution method	*F. verticillioides*	Not stated	Not stated	Inhibition of 73.96% at 1 µL/mL	[117]
		*F. oxysporum*			Inhibition of 85.56% at 1 µL/mL	
*Mentha piperita* L. (Lamiaceae)	microbroth dilution assay	*F. oxyporum* (MNHN 963917)	Amphotericin	MIC value of 1.50 µg/mL	MIC value of 1.50 µg/mL	[197]
		*F. acuminatum*		MIC value of 1.50 µg/mL	MIC value of 2.50 µg/mL	
		*F. solani*		MIC value of 1.25 µg/mL	MIC value of 10.0 µg/mL	
		*F. tabacinum*		MIC value of 1.35 µg/mL	MIC value of 1.50 µg/mL	
*Pimenta dioica* (L.) Merr. (Myrtaceae)	agar dilution method	*F. oxysporum*	Not stated	Not stated	Inhibition of 100% at 1 µL/mL	[155]
		*F. verticillioides*			Inhibition of 100% at 1 µL/mL	
*Pimpinella anisum* L. (Apiaceae)	broth microdilution method	*F. solani*	Itraconazole; Fluconazole; Ketoconazole	7; 18; 12 µg/mL	MIC value of 85 µg/mL	[183]
		*F. oxysporum*		9; 10; 9 µg/mL	MIC value of 120 µg/mL	
*Rosa damascena* P. Mill. (Rosaceae)	microdilution technique	*F. subglutinans*	fluconazole	Not stated	MIC value of 0.62 mg/mL	[188]
		*F. solani*			MIC value of 0.29 mg/mL	
		*F. tricinctum*			MIC value of 0.14 mg/mL	
		*F. sporotrichioides*			MIC value of 0.29 mg/mL	
		*F. verticillioides*			MIC value of 0.14 mg/mL	
		*F. oxysporum*			MIC value of 0.29 mg/mL	
		*F. semitectum*			MIC value of 0.64 mg/mL	
		*F. equiseti*			MIC value of 0.30 mg/mL	
*Rosmarinus officinalis* (rosemary) (Lamiaceae)	broth microdilution method	*F. solani*	Itraconazole; Fluconazole; Ketoconazole	7; 18; 12 µg/mL	MIC value of 320 µg/mL	[183]
		*F. oxysporum*		9; 10; 9 µg/mL	MIC value of 410 µg/mL	
*Salvia sclarea* L. (Lamiaceae)	agar dilution method	*F. oxysporum*	Not stated	Not stated	Inhibition of 58.82% at 1 µL/mL	[155]
		*F. verticillioides*			Inhibition of 65.09% at 1 µL/mL	
*Satureja hortensis* L. (Lamiaceae)	microdilution technique	*F. subglutinans*	fluconazole	Not stated	MIC value of 0.95 mg/mL	[188]
		*F. solani*			MIC value of 0.14 mg/mL	
		*F. tricinctum*			MIC value of 0.14 mg/mL	
		*F. sporotrichioides*			MIC value of 0.27 mg/mL	
		*F. verticillioides*			MIC value of 0.14 mg/mL	
		*F. oxysporum*			MIC value of 0.14 mg/mL	
		*F. semitectum*			MIC value of 0.14 mg/mL	
		*F. equiseti*			MIC value of 0.62 mg/mL	
*Schinus molle* L. (Anacardiaceae)	disc diffusion method	*F. verticillioides*	Not stated	Not stated	IC_50_ of 1226.76 µL/L	[158]
*Silene armeria* L. (Caryophyllaceae)	disc diffusion method	*F. oxysporum* KACC 41083	Not stated	Not stated	MIC value of 500 µg/mL	[198]
		*F. solani* KACC 41092			MIC value of 125 µg/mL	
*Stachys pubescens* Ten. (Lamiaceae)	broth microdilution method	*F. oxysporum*	amphotericin B	0.5 µg/mL	MIC value of 1 µg/mL	[185]
*Syzigium aromaticum* L. (Myrtaceae)	toxic medium assay	*F. oxysporum isolate* S-1187.	ICA-Thiabendazole^®^ 500SC	Not stated	Inhibition of 83% at 250 µL/L	[186]
*Tagetes riojana* M. Ferraro Biurrum 8753 (Asteraceae)	disc diffusion method	*F. verticillioides*	Not stated	Not stated	IC_50_ of 764.75 µL/L	[158]
*Thymus daenensis* Celak. (Lamiaceae)	broth microdilution method	*F. oxysporum*	amphotericin B	0.5 µg/mL	MIC value of 4 µg/mL	[185]
*Thymus kotschyanus* Boiss. & Hohen. (Lamiaceae)	broth microdilution method	*F. oxysporum*	amphotericin B	0.5 µg/mL	MIC value of 0.5 µg/mL	[185]
		*F. solani*	Itraconazole; Fluconazole; Ketoconazole	7; 18; 12 µg/mL	MIC value of 40 µg/mL	[183]
		*F. oxysporum*		9; 10; 9 µg/mL	MIC value of 75 µg/mL	
*Thymus mastichina* L. (Lamiaceae)	agar dilution method.	*F. verticillioides*	Not stated	Not stated	Inhibition of 51.48% at 1 µL/mL	[155]
*Thymus vulgaris* L. (Lamiaceae)	microdilution technique	*F. solani*	fluconazole	Not stated	MIC value of 0.16 mg/mL	[188]
		*F. tricinctum*			MIC value of 0.19 mg/mL	
		*F. sporotrichioides*			MIC value of 0.61 mg/mL	
		*F. verticillioides*			MIC value of 0.14 mg/mL	
		*F. oxysporum*			MIC value of 0.14 mg/mL	
		*F. semitectum*			MIC value of 0.19 mg/mL	
		*F. equiseti*			MIC value of 0.98 mg/mL	
*Thymus vulgaris* L. (Lamiaceae)	toxic medium assay	*F. oxysporum isolate* S-1187.	ICA-Thiabendazole^®^ 500SC	Not stated	Inhibition of 61% at 250 µL/L	[186]
*Thymus vulgaris* L. (Lamiaceae)	agar dilution method	*F. culmorum*	Not stated	Not stated	Inhibition of 99.71% at 1 µL/L	[196]
*Thymus vulgaris* L. (Lamiaceae)	agar dilution method	*F. oxysporum*	Not stated	Not stated	Inhibition of 98.41% at 1 µL/mL	[155]
		*F. verticillioides*			Inhibition of 98.22% at 1 µL/mL	
*Xylopia aethiopica* (Dunal) A. Rich. (Annonaceae)	incorporation method	*F. oxysporum*	Not stated	Not stated	MIC value of 3000 ppm	[199]
*Zataria multiflora* Boiss. (Lamiaceae)	broth dilution method	*F. solani isolates*	Not stated	Not stated	MIC value of 76 µg/mL	[189]
		*F. oxysporum isolates*			MIC value of 66 µg/mL	
		*F. verticillioides isolates*			MIC value of 77 µg/mL	
		*F. poae isolates*			MIC value of 99 µg/mL	
		*F. equiseti isolates*			MIC value of 99 µg/mL	
*Zataria multiflora* Boiss. (Lamiaceae)	broth microdilution method	*F. solani*	Itraconazole; Fluconazole; Ketoconazole	7; 18; 12 µg/mL	MIC value of 40 µg/mL	[183]
		*F. oxysporum*		9; 10; 9 µg/mL	MIC value of 20 µg/mL	
*Zhumeria majdae* Rech. f. & Wendelbo (Lamiaceae)	disk diffusion method	*F*. *graminearum*	Not stated	Not stated	Inhibition of 75.11% at 1000 µL/L	[192]
		*F*. *asiaticum*			Inhibition of 100% at 1500 µL/L	
		*F*. *redolens fsp. dianthus*			Inhibition of 100% at 1500 µL/L	
		*F*. *verticillioides*			Inhibition of 70.66% at 1500 µL/L	
		*F*. *oxysporum f. sp. lentis*			Inhibition of 60.44% at 1500 µL/L	
*Zingiber cassumunar* Roxb. (Zingiberaceae)	agar dilution method	*F. verticillioides*	Not stated	Not stated	Inhibition of 67.46% at 1 µL/mL	[155]

**Table 4:** Antifungal activity of compounds isolated from plants used in traditional medicine. The compounds were evaluated against different *Fusarium* pathogens and their antifungal activities were reported as minimum inhibitory concentration, percentage inhibition or half-maximal effective concentration.

Compound	Chemical Structure	Plant Species (Family)	Plant Part	Organism Tested	Positive Control	Activity of Positive Control	Results	Reference
(±)-Qinghaocoumarin A	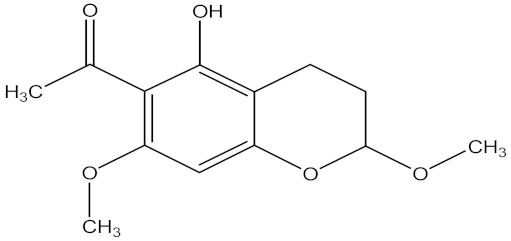	*Artemisia annua* L. (Asteraceae)	leaves	*F. oxysporum*	Hymexazol	13.02 µg/mL	MIC value of 18.75 µg/mL	[200]
				*F. solani*		41.67 µg/mL	MIC value of 18.75 µg/mL	
(3*R*,3*aS*,6*R*,6*aS*,7*aR*,8*aS*,9*aS*,9*bR*)-decahydro-9*b*-hydroxy-3,6,8*a*-trimethyl-oxireno[*c*]pyrano [4,3,2-*jk*] benzoxepin-2(3*H*)-one	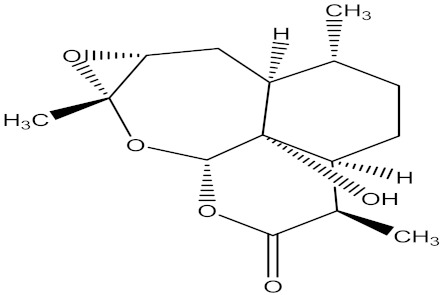			*F. oxysporum*	Hymexazol	13.02 µg/mL	MIC value of 62.50 µg/mL	
				*F. solani*		41.67 µg/mL	MIC value of 21.79 µg/mL	
1,2-dimethoxy-4(2-propenyl) benzene	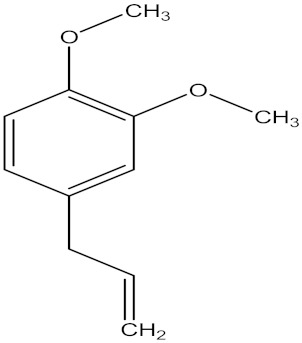	*Acorus tatarinowii* Schott (Acoraceae)	whole plant	*F*. *oxysporum f. sp. niveum*	Not stated	Not stated	Inhibition of 100% at 0.4 g/L	[201]
3,4-dihydroxy-3,4-dimethoxy-6,7- cyclolignan	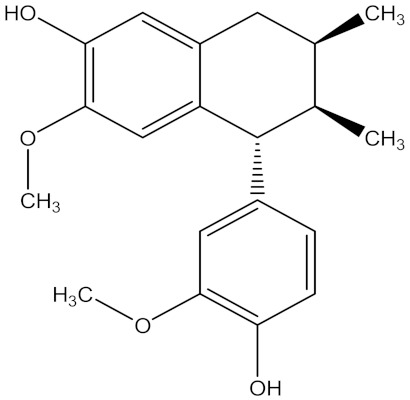	*Larrea divaricata* Cav. (Zygophyllaceae)	leaves and stem	*F. verticillioides*	Not stated	Not stated	MIC value of 250 µg/mL	[202]
				*F. graminearum*			MIC value of 15.6 µg/mL	
				*F. solani*			MIC value of 125 µg/mL	
5-hydroxy-7,40-dimethoxyflavone	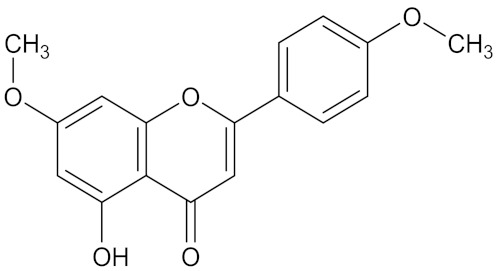	*Combretum erythrophyllum* (Burch.) Sond. (Combretaceae)	leaves	*F. verticilloides*	amphotericin B	0.003 mg/mL	0.31 mg/mL	[203]
				*F. proliferatum*		0.0004 mg/mL	0.01 mg/mL	
				*F. solani*		1.2 mg/mL	0.31 mg/mL	
				*F. graminearum*		2.3 mg/mL	0.63 mg/mL	
				*F. chlamydosporum*		2.3 mg/mL	0.63 mg/mL	
3′, 4′-de- O-methylenehinokinin	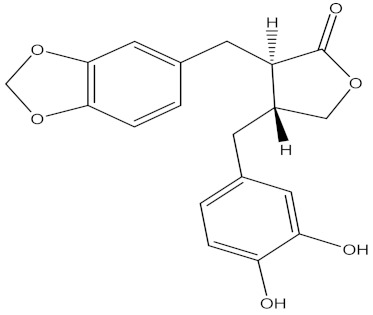	*Artemisia annua* L. (Asteraceae)	leaves	*F. oxysporum*	Hymexazol	13.02 µg/mL	MIC value of 31.25 µg/mL	[200]
				*F. solani*		41.67 µg/mL	MIC value of 75.00 µg/mL	
3α,7α-dihydroxy amorph-4-ene 3-acetate	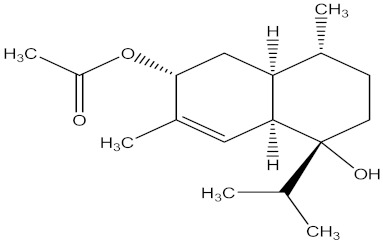		leaves	*F. oxysporum*	Hymexazol	13.02 µg/mL	MIC value of 50.00 µg/mL	
				*F. solani*		41.67 µg/mL	MIC value of 43.75 µg/mL	
artemetin	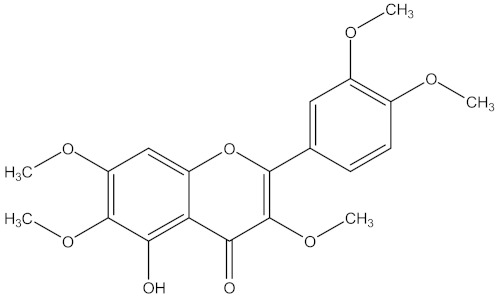			*F. oxysporum*		13.02 µg/mL	MIC value of >150.00 µg/mL	
				*F. solani*		41.67 µg/mL	MIC value of >150.00 µg/mL	
dehydrodiconiferyl alcohol	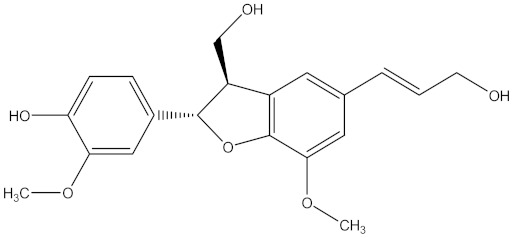			*F. oxysporum*		13.02 µg/mL	MIC value of 150.00 µg/mL	
				*F. solani*		41.67 µg/mL	MIC value of 37.50 µg/mL	
denudatin A	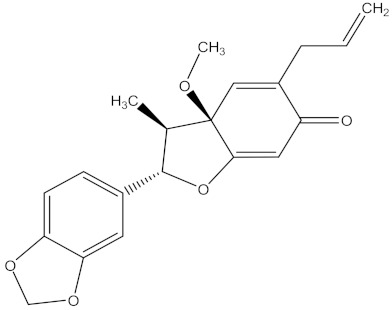			*F. oxysporum*		13.02 µg/mL	MIC value of 150.00 µg/mL	
				*F. solani*		41.67 µg/mL	MIC value of 37.5 µg/mL	
denudatin B	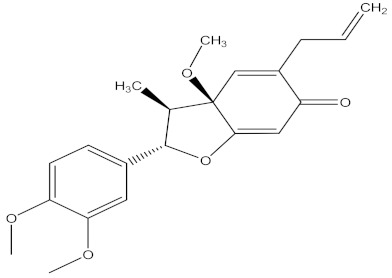			*F. oxysporum*		13.02 µg/mL	MIC value of 37.50 µg/mL	
				*F. solani*		41.67 µg/mL	MIC value of 87.5 µg/mL	
futokadsurin B	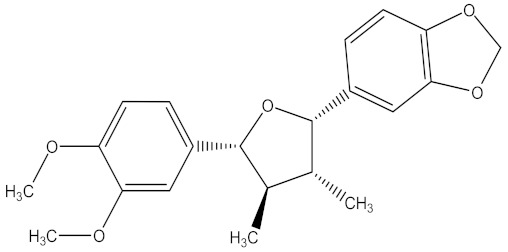			*F. oxysporum*		13.02 µg/mL	MIC value of 150.00 µg/mL	
				*F. solani*		41.67 µg/mL	MIC value of 75.00 µg/mL	
futokadsurin C	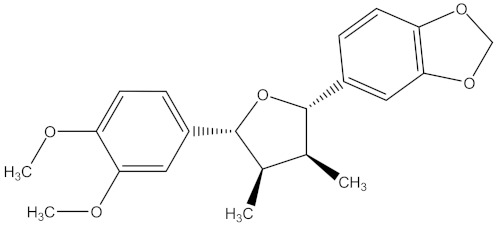			*F. oxysporum*		13.02 µg/mL	MIC value of 125.00 µg/mL	
				*F. solani*		41.67 µg/mL	MIC value of 100.00 µg/mL	
Gallic acid	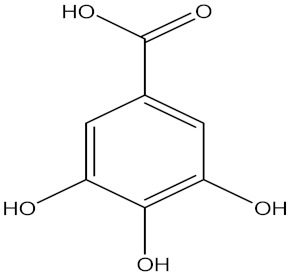	*Terminalia nigrovenulosa* Pierre (Combretaceae)	bark	*F. solani*	Not stated	Not stated	Inhibition of 75% at 500 ppm	[204]
Maslinic acid	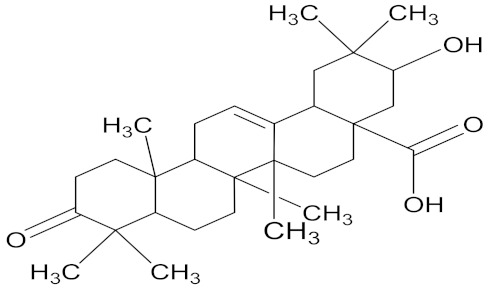	*Combretum erythrophyllum* (Combretaceae)	leaves	*F. oxysporum*	amphotericin B	1.2 mg/mL	0.31 mg/mL	[203]
				*F. verticilloides*		0.003 mg/mL	0.08 mg/mL	
				*F. subglutinans*		9.4 mg/mL	0.63 mg/mL	
				*F. proliferatum*		0.0004 mg/mL	0.31 mg/mL	
				*F. solani*		1.2 mg/mL	0.63 mg/mL	
				*F. graminearum*		2.3 mg/mL	0.63 mg/mL	
*N*_1_-decarbomethoxy chanofruticosinic acid	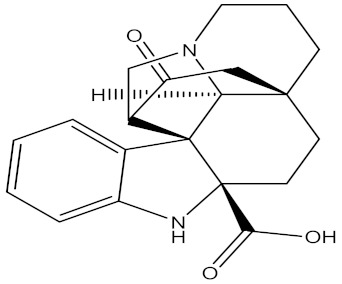	*Kopsia hainanensis* Tsiang (Apocynaceae)	leaves and stem	*F. oxysporum f. sp. Cubense*	mildothane	EC50 value of 57.0 µg/mL	EC_50_ value of 15.2 µg/mL	[205]
				*Fusarium oxysporum f. sp. Niveum*		EC50 value of 101.0 µg/mL	EC_50_ value of 43.8 µg/mL	
							EC_50_ value of 31.8 µg/mL	
nordihydroguaiaretic acid	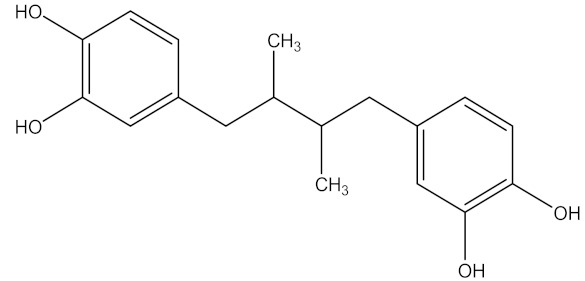	*Larrea divaricata* Cav. (Zygophyllaceae)	leaves and stem	*F. graminearum*	Not stated	Not stated	MIC value of 62.5 µg/mL	[202]
				*F. solani*			MIC value of 250 µg/mL	
				*F. verticillioides*			MIC value of 125 µg/mL	
penduletin	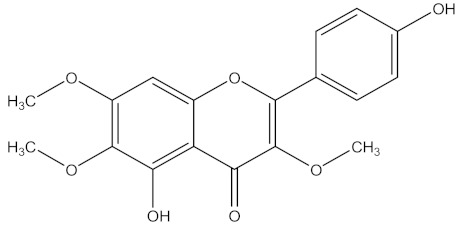	*Artemisia annua* L. (Asteraceae)	leaves	*F. oxysporum*	Hymexazol	13.02 µg/mL	MIC value of 100.00 µg/mL	[200]
				*F. solani*		41.67 µg/mL	MIC value of 100.00 µg/mL	
Phloroglucinol derivative	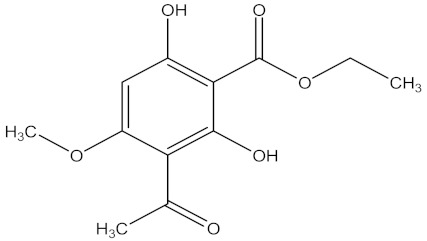			*F. oxysporum*		13.02 µg/mL	MIC value of 62.50 µg/mL	
				*F. solani*		41.67 µg/mL	MIC value of 87.50 µg/mL	
Qinghaocoumarin B	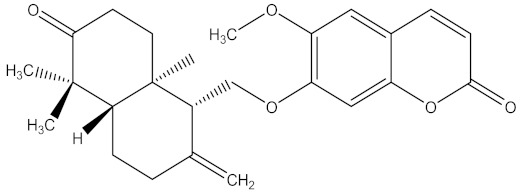			*F. oxysporum*		13.02 µg/mL	MIC value of 62.50 µg/mL	
				*F. solani*		41.67 µg/mL	MIC value of 43.75 µg/mL	
Withaferin A	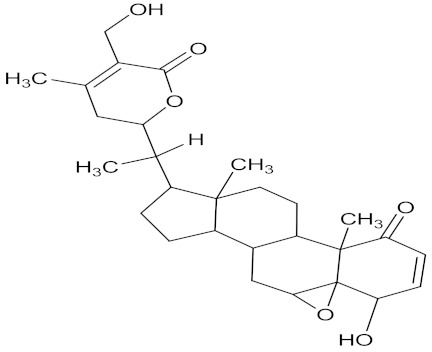	*Withania somnifera* (L.) Dunal. (Solanaceae)	leaves	*F. verticilloides*	amphotericin B	0.003 mg/mL	0.16 mg/mL	[203]
Qinghaolignan B	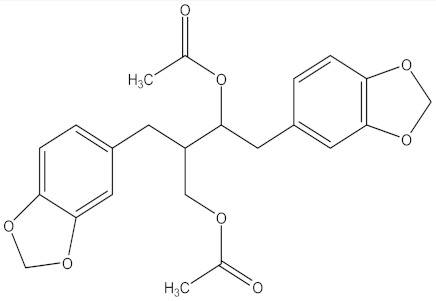	*Artemisia annua* L. (Asteraceae)	leaves	*F. oxysporum*	Hymexazol	13.02 µg/mL	MIC value of 150.00 µg/mL	[200]
				*F. solani*		41.67 µg/mL	MIC value of 37.50 µg/mL	

## 4. Mechanisms of Action

Understanding the mechanisms of action of natural products from medicinal plants or synthetic chemicals against *Fusarium* pathogens is an important approach towards crop disease control. Pesticides inhibit the growth of pathogens by interfering with numerous useful metabolic processes of the pathogens. As an example, benzimidazole fungicides were reported to inhibit fungi by binding protein subunits of spindle and disrupting their functions [101]. Additionally, the application of pesticides may activate morphological and biochemical defence mechanisms of the crop against diseases. Although no mechanism of actions was proposed, studies have reported treatment of tomato plants with chemicals such as K- and Na-benzthiazolylthiocycloate, 4-chloro-3,5-dimethylphenoxyethanol, dinitroaniline and DL-3-aminobutyric acid, which induce the plant defence mechanism against *Fusarium* wilt disease [206]. The different mechanisms of action of fungicides acting against *Fusarium* pathogens are summarized in Table 5.

Generally, antifungal chemicals inhibit pathogen growth by interfering with the biosynthesis of the major components of the cell wall and cell membrane or through the formation of ion channels on the cellular membrane [207,208]. Antifungal agents can act by inhibiting normal functions of the topoisomerase enzymes, increasing permeability of fungal cell wall and by targeting the plasma membrane in most pathogens [209]. With regard to plant products (extracts and essential oils), their main mechanisms of action can include the following: disruption of the fungal cell wall integrity through the inhibition of chitin and β-glucans synthesis; disruption of the cell membrane, such as by binding to or inhibiting ergosterol biosynthesis; mitochondria dysfunction arising from inhibition of electron transport and respiratory chain proton pumps; cell division inhibition via interference with microtubule polymerization; inhibition of ribonucleic acid, deoxyribonucleic acid or protein synthesis; and efflux pump inhibition [210]. Disruption of the fungal membrane may lead to membrane permeability and eventually prevent normal biochemical functions [211]. Nonetheless, more studies are required in order to fully understand the different mechanisms of actions and their dynamics, particularly of medicinal plant products (extracts, essential oils and isolated compounds).

**Table 5:** Possible mechanisms of action of pesticides against *Fusarium* phytopathogenic species.

Extracts/Fungicides	Target Site	Possible Mechanism of Action	Reference
95% ethanol extract of *Curcuma longa* (Zingiberaceae)	Protein synthesis and enzymatic pathways	Inhibition of GAPDH, tRNA synthetase family II and Zinc binuclear structural-containing fungal protein	[212]
	Cell membrane synthesis	Inhibition of ergosterol synthesis	
	Respiratory system	Suppression of the activity of NADH oxidase and SDH	
2,5-dicyclopentylidene cyclopentanone	Cell membrane and cell wall	Inhibition of sterol biosynthesis	[213]
Amoxicillin, Chloramphenicol, Erythromycin and Raficillin	Cell wall enzymatic pathways	Inhibit the polygalacturonase and pectinmethylgalacturonase enzyme activities	[209]
Rifampin and Rifabutin, members of the Rifamycin class and Azithromycin	Protein synthesis	Inhibition of RNA and protein synthesis	[214,215,216]
Benzimidazole	Protein synthesis	Binding to fungal β-tubulin and disrupt microtubule dynamic including interference with monomeric tubulin polymerization	[217]
Peptide Fengycins	Cell membrane	Formation of ion channels on cellular membrane by interfering with synthesis of ergosterol	[208]
Azole fungicides	Fungal cell membrane	Inhibition of the heme protein and 14α-demethylation of lanosterol	[218]

## 5. Challenges and Future Perspectives

There is an abundance of medicinal plant species that can be screened for antifungal activity of their extracts, essential oils and isolated compounds as potential biocontrol agents for possible application in crop production. The number of in vitro antifungal activity studies of medicinal plant materials against human and crop pathogens is increasing every year [219,220,221]. On the other hand, the number of formulated products developed from these natural resources remains very few in comparison. Many researchers in academic and research institutions are very interested in evaluating medicinal plant materials for application as safe and biodegradable pesticides. As shown in Table 2, Table 3 and Table 4, these natural products have exhibited very good antifungal activity against different *Fusarium* pathogens; however, there are challenges and limitations that must be addressed in order to develop these natural resources into beneficial final products or biopesticides.

It is critical that appropriate valid test assays incorporating suitable positive and negative controls be used for in vitro screening. The results should include the minimum inhibitory concentration that allows for effective inter-laboratory comparisons of the results. Biological activities of crude extracts, essential oils and isolated compounds are generally dose-dependent activities. Hence, while stating the inhibition percentage at a concentration may indicate potency at that concentration, it does not allow for an effective comparison at dose-dependent levels. It is desirable that the assays also determine the potential fungicidal effect of the extracts and/or compounds. Many plant extracts have demonstrated potent antifungal activity (with MIC values below 1.0 mg/mL) using in vitro assays (Table 2), but only a few were tested in vivo [130,222,223,224,225,226,227]. The potent in vitro antifungal activity of *Melia azedarach*, *Combretum erythrophyllum* and *Quercus acutissima* leaf extracts [130] were confirmed in vivo. The leaf extract of *Melia azedarach* showed strong antifungal activity against *F. proliferatum* inoculated on maize seeds, while combined leaf extracts from *Combretum erythrophyllum* and *Quercus acutissima* exhibited potent inhibitory activity against *F. verticilloides* in vivo without any phytotoxic effect [130]. One of the limiting factors is the unavailability of resources and skills required to conduct relevant in vivo experiments either in the greenhouse or in the field. This gap can, however, be bridged through collaborative research. The frustrating and time-consuming process and regulations involved during registration of biopesticides is also a challenge. The amount of plant extracts, essential oils or isolated compounds required to conduct in vivo field experiments can be a limiting factor, especially if these are obtained from non-renewable plant parts. Thus, we recommend that the use of renewable plant parts such as the leaves be given more attention in designing appropriate experiments. Medicinal plants with very promising antifungal activity against crop pathogens may need to be cultivated in order to guarantee a regular supply of quality raw materials required for product development. Quality control protocols and the standardization of cultivation practices for selected plants are important to ensure consistent high-quality raw materials [228]. On the other hand, the use of invasive species such as those in the Solanaceae family that demonstrate potent in vitro activity, if confirmed in vivo, may be a relatively cheap alternative.

Several studies have focused on individual plant extracts (Table 2), essential oils (Table 3) or isolated compounds (Table 4) against some specific pathogens. In some cases, the antifungal activity demonstrated by an isolated compound may be disappointing when compared to the originating plant extracts or fractions [229]. Although pathogen and plant species specific, it was noticed that combinations of extracts from different plant species may improve antifungal activity [131]. In a study evaluating the antifungal effect of combining plant extracts against *Fusarium* species, 150 extract out of 204 extract combinations exhibited either a synergistic or additive effect [131]. In particular, a combination of *Harpephyllum caffrum* and *Combretum erythrophyllum* leaf acetone extracts demonstrated very strong synergistic inhibitory activity in comparison to their individual extracts against *F. graminearum*, *F. proliferatum* and *F. verticillioides* [131]. Plants contain several metabolites that could interact in various ways to produce desired activities against a panel of microorganisms. The desired activity may therefore be lost when isolated compounds acting together in a synergistic manner in an extract are tested individually [230]. It may be worthwhile to evaluate the potentiating effect of different combinations of plant extracts or isolated compounds in vitro and in vivo as part of the screening process for formulating plant-based products. The phytotoxicity determination and potential biostimulant effect of promising extracts and/or compounds on plant growth as well as their biochemical mode of action need to be established.

Ordinarily, plant extracts, essential oils and isolated compounds obtained from medicinal plants are poorly soluble in water. Products or formulations prepared from these plant materials are usually dissolved in organic solvents and that itself poses a toxicity challenge. Such organic solvents may be phytotoxic to the crops and can also evaporate during storage period, thus affecting the concentration of the constituents. Furthermore, the formulation or product may not persist in the environment to deliver desired effect and may lead to frequent biopesticide applications [80]. Some of these challenges may be addressed through application and implementation of nanotechnology strategies, which can improve the stability and efficacy of natural products (extracts, essential oils and isolated compounds) developed from medicinal plants.

There must be robust analytical techniques and quality control procedures to determine chemical composition and quantity of active ingredients in both raw materials and finished products. Agronomical practices and post-treatment processes, including drying, processing and storage, have a negative impact on the activity and phytochemical content of plant extracts. These practices were reported to be plant species specific and may affect the quality of plant products [231,232,233]. In addition, the chemical structures of isolated compounds that exhibited good antifungal activity against *Fusarium* may be used as scaffold molecules or in computational studies for designing synthetic approaches that will result in more yield during industrial production. Different derivatives for those active compounds may also be developed.

The use of nanotechnology is an important step towards development of biopesticides from natural products. The combination of nanoparticles into a delivery system of natural plant products was used in several studies to increase therapeutic activity, bioavailability and target a specific action site of the product. This application is well known and has been successful in the treatment of human diseases [234,235]. A similar approach may be applied in crop protection to increase stability and activity of plant extracts. Currently there is a paucity of information on the incorporation of nanotechnology strategies in order to improve stability and efficacy of natural products from plants with potential for controlling crop diseases in the agricultural sector. Although formulation development may add cost to the overall process, this field of research is worth investigating.

With regard to essential oils, which are a mixture of different volatile compounds, their screening process should include their chemical profiles. Thereafter, the structure-activity of the oils can be computed to establish which chemical constituent(s) demonstrated stronger antifungal activity. That information can be utilized to specifically synthesize such active compounds. The constituents or compounds may be combined into different ratios and re-evaluated for antifungal activity and further developed into a product. The phenomenon of combining different constituents from essential oils may also be done with isolated active compounds. This approach may help to delay development of fungal resistance.

Regardless of the time-consuming procedures required to develop and register biopesticide products, it is important to carefully study and evaluate efficacy, safety and stability of natural plant products. This will help to have a better understanding of their toxicity towards non-target organisms and their long-term impact on the environment. In vivo cytotoxicity determination and mechanisms of action of these natural products against tested *Fusarium* pathogens are other areas of study to be explored. In conjunction with stability studies, the knowledge of their cytotoxicity, phytotoxicity and mechanisms of action would make it easy to also understand their frequency of application in the field when combating crop disease outbreaks.

## 6. Conclusions

To address the challenges of pesticide resistance development, as evidenced by most *Fusarium* pathogens against conventional synthetic pesticides, natural products from medicinal plant species are considered as alternative control agents. Extracts from plant species in the families Solanaceae, Combretaceae and Fabaceae are among the most commonly used agents against *Fusarium* pathogens. Other families with a high potential include the Euphorbiaceae, Rubiaceae, Asteraceae and Celastraceae families. The majority of studies have focused attention on the use of leaves, a renewable plant part, as the source of secondary metabolites with antifungal activity against *Fusarium* pathogens. While different organic solvents have been used for extraction of bioactive compounds as crude extracts, water extract demonstrated relatively good antifungal activity in some cases. Water is readily available and may be used by resource-poor farmers for extraction. On the other hand, the extraction of plant materials with organic solvents, such as acetone and ethyl acetate, enhances the possibility of extracting a wide range of antifungals. Essential oils derived from species belonging to the Lamiaceae, Apiaceae, Asteraceae and Myrtaceae families demonstrated potent activity against *Fusarium* pathogens. Particularly noteworthy are the essential oils from *Thymus vulgaris*, *Cymbopogon citratus* and *Melaleuca alternifolia*. Medicinal plant products (extracts, essential oils and isolated compounds) are perceived to be safer, are biodegradable and are environmentally friendly. They are also expected to have less side effects since they have been used in many countries to treat different aliments affecting animals and human. Plant products are inherently unstable to higher temperatures and sunlight; therefore, they may not persist in the environment for a very long period of time. Incorporation of nanotechnology approaches may be used to improve stability and efficiency of natural products developed from medicinal plants. Medicinal plants are abundant sources of different bioactive metabolites or chemicals. Therefore, investment in the development of medicinal plant products to control crop diseases including those caused by *Fusarium* pathogens is a growing sector to be closely considered. Regardless of the challenges, plant natural products remain potential alternative sources of environmentally friendly biopesticides to control *Fusarium* pathogens known to cause diseases in crop production.
